# Subject-specific modeling framework for particle deposition using computational fluid dynamics

**DOI:** 10.1016/j.jaerosci.2025.106660

**Published:** 2025-08-18

**Authors:** Ignacio R. Bartol, Martin S. Graffigna Palomba, Robert J. Dawson, Wesley E. Bolch, Mauricio E. Tano, Shaheen A. Dewji

**Affiliations:** a Nuclear and Radiological Engineering and Medical Physics Programs, George W. Woodruff School of Mechanical Engineering, Georgia Institute of Technology, 770 State St NW, Atlanta, 30332-0405, GA, United States of America; b Collaborative Computing Center (C3), Idaho National Laboratory, 955 MK Simpson Boulevard, Idaho Falls, 83415, ID, United States of America; c J. Crayton Pruitt Family Department of Biomedical Engineering, University of Florida, 1275 Center Drive, Gainesville, 32611-6550, FL, United States of America

**Keywords:** Subject-specific modeling, Particle deposition, Automated workflow, Computational fluid and particle dynamics, Human airways, Monte carlo radiation transport, Computer vision, ICRP, MPPD, Inhalation dosimetry, Aerosol dosimetry

## Abstract

Quantifying particle deposition and dose in the respiratory tract requires a physiologically realistic representation and reproducible computational workflows. However, existing modeling frameworks, such as the International Commission on Radiological Protection (ICRP) compartmental models and the Multiple Path Particle Dosimetry (MPPD) tool, lack detailed deposition profiles and subject-specific capabilities. The combination of advances in computer vision algorithms applied to the respiratory tract and Computational Fluid and Particle Dynamics (CFPD) allows high-fidelity simulations of particle behavior in anatomically accurate geometries derived from individual CT scans. The segmentation, preprocessing, and file preparation task for a CFPD simulation was often time-consuming, and no prior studies to-date have yet presented a fully automated framework.

This work presents a fully automated workflow to obtain individualized particle deposition profiles in the human respiratory tract. The pipeline starts with segmenting upper and lower airway geometries using morphological and deep learning-based methods, generating three-dimensional (3D) models from CT imaging data. Next, a series of algorithms are presented to quality check and prepare the 3D geometry for a CFD or CFPD simulation. The preprocessing step includes correcting geometric artifacts, enforcing a physically consistent mesh, and automatically identifying and capping multiple outlets, which is required for CFD/CFPD simulations. These processed models are then input into open-source (OpenFOAM) or commercial (StarCCM+) CFD solvers, where flow and transient particle transport equations — including turbulence and particle–wall interactions are solved under realistic breathing conditions. Finally, the resulting particle deposition profiles can be integrated with Monte Carlo radiation transport codes and state-of-the-art computational phantoms to assess organ-specific absorbed doses in scenarios of radioactive aerosol inhalation.

The presented work streamlines respiratory tract segmentation, preprocessing for CFD/CFPD simulations, and integration with dose assessment workflows, reducing manual intervention and improving access to high-fidelity, subject-specific modeling. The high precision in predicted particle deposition and dose distributions can improve personalized treatment strategies in respiratory medicine and refine dose estimates for radiation protection.

## 1. Introduction

Assessing aerosol dosimetry from inhaled airborne solid particles is relevant to a range of applications, spanning radiation protection, therapeutic drug delivery, and environmental health. The prevailing framework for these assessments has been largely based on mathematical models developed by the International Commission on Radiological Protection (ICRP), specifically detailed in Publications 66 and 130 (ICRP, 1994; Paquet et al., 2015). The ICRP models conceptualize the human respiratory tract (HRT) as a series of simplified compartments, each assumed to have uniform particle deposition profiles (PDPs). While these models have been foundational in establishing guidelines for radiation protection by incorporating scaling factors that account for particle size distributions (Bolch et al., 2001), they inherently lack individualized physiological specificity. Parameters such as tracheal diameter, lung volume, bronchi length, and subject-specific geometric features are generalized, which limits the precision of dosimetric assessments for individuals whose respiratory anatomy deviates from the population average.

Aerosol dosimetry models have evolved through different conceptual frameworks and regulatory agencies. Before the ICRP compartmental approach, significant contributions were done by the National Council on Radiation Protection and Measurements (NCRP) model introduced in 1997 (National Council on Radiation Protection and Measurements, 1997). The NCRP Report No. 125, developed a model that was primarily based on the earlier work of Yeh and Schum (1980b), contrasting the model on ICRP Publication 66, which was grounded in the approach by Egan et al. (1989). Yeh and Schum's model utilized detailed airway branching structures and deposition efficiencies derived from experimental data in a silicone rubber replica of the human tracheobronchial tree. Considering deformation due to gravity in the model, the authors computed aerosol transport and deposition within the respiratory tract using theoretical estimations. Conversely, Egan's model and its subsequent adaptation by ICRP emphasized new experimental work with volunteers, in addition to simplified compartments and empirical deposition mechanisms, allowing broader population-based assessments by incorporating anatomical data in their equations. While both the NCRP and ICRP models have been widely used, each has limitations in representing subject-specific morphological details of the tracheobronchial tree, with the NCRP model providing relatively detailed airway morphology but still lacking individualized variability.

These limitations stem from the use of compartment-based representations that generalize airway branching and morphology, failing to capture localized deposition patterns resulting from flow asymmetry or pathological conditions. The HRT is divided in two main sections, the extrathoracic (ET) and intrathoracic regions. The ET region, is comprised by the nostrils, nasal passages, larynx and pharynx. On the other hand, the intrathoracic airways are divided into the tracheobronchial (TB) region, comprising the trachea (G0), bronchus (G1), bronchi, and bronchioles up to the 15th generation (G15). The pulmonary region is comprised by the terminal bronchioles (G16-G17) and alveolar sacs (G18 to G23), where gas exchange to the lungs takes place, as discussed in Kleinstreuer and Zhang (2010). ICRP Publication 66 introduced the first comprehensive mathematical model of aerosol deposition in the HRT by dividing the respiratory tract into five distinct regions: the extrathoracic (ET1 and ET2), bronchial (BB), bronchiolar (bb), and alveolar-interstitial (AI) regions. Deposition mechanisms such as impaction, sedimentation, and diffusion are modeled using empirical equations that consider particle properties (size, density, and aerodynamic diameter) and generalized anatomical and physiological parameters (ICRP, 1994). Particle deposition fractions in each region are determined using average airway dimensions, breathing patterns, and airflow rates. ICRP Publication 130 builds upon this framework by refining some of the assumptions in ICRP Publication 66, particularly for ultrafine and nano-sized particles. However, the basic structure of compartmental modeling remains unchanged, except for some structures previously included in the ET1 region that are now included in the ET2 region in the updated ICRP Publication 130 model (Paquet et al., 2015). Both models use population-averaged data to estimate deposition, scaling these estimates for different age groups and physical activities. However, these models cannot account for subject-specific anatomical variations.

The primary limitations of ICRP models arise from their simplified, compartmental representations of the TB and PU regions. Parameters such as tracheal diameter, bronchial branching angles, and alveolar geometry are generalized, ignoring the significant inter-subject variability that can influence aerosol deposition patterns (Bolch et al., 2001). Differences in lung morphology between a healthy and diseased lung are not adequately captured. Furthermore, the assumption of uniform deposition within each compartment fails to account for localized hotspots of deposition that occur due to airflow turbulence, anatomical asymmetry, or different pathological conditions.

A more detailed dosimetry model that incorporates a variety of respiratory tract models and a range of tunable physiological parameters is the Multiple Path Particle Dosimetry model (MPPD) introduced by Price et al. (2002) and updated later by Miller et al. (2016); the MPPD model allows for detailed customization of physiological and aerodynamic parameters. Unlike the compartmentalized approach of ICRP models, MPPD leverages a more sophisticated framework to account for the intricate airway geometry and inter-subject variability. Furthermore, the MPPD software is built on a robust mathematical framework that uses a combination of symmetric and asymmetric lung geometries to represent the branching structure of the human respiratory tract. The available geometries in MPPD range in complexity, from simplified Weibel-like dichotomous models to more complex, asymmetric structures that mimic anatomical variability across different lung generations. The model divides the respiratory tract into the extrathoracic, tracheobronchial, and alveolar regions, allowing for region-specific predictions of particle deposition. Moreover, users can tune a wide array of physiological parameters, including adult and pediatric lung geometries representing ages from 3 months to 21 years old, respiratory parameters (e.g., tidal volume, flow rate, and breathing frequency), and particle characteristics (e.g., size, density, distribution, and shape) among others. Additionally, MPPD incorporates particle interaction mechanisms such as impaction, sedimentation, and diffusion, supporting modeling of aerosol deposition, clearance, and retention ranging from nanometers to micrometers scale. MPPD is based on previous theoretical and experimental derivations of deposition efficiencies in their calculations (Asgharian et al., 2001).

Another feature of the MPPD model is its flexibility in simulating various breathing scenarios, including nasal and oral breathing, resting and exertion states, and age-specific respiratory profiles. This adaptability makes MPPD suitable for exploring how different populations, such as children and adults, respond to airborne particle exposure. Moreover, the MPPD model allows users to define and test multiple aerosol compositions and environmental conditions, such as humidity and temperature, which can affect particle behavior. Through these tunable parameters, MPPD has supported progress in aerosol science, toxicology, and radiation dosimetry, addressing limitations inherent in more generalized ICRP models.

While ICRP and MPPD can provide fast and efficient particle dosimetry results, the models cannot accommodate subject-specific respiratory tract geometries due to the nature of the models. In the context of modern healthcare and environmental studies, there is an increasing demand for high-resolution, subject-specific data to improve the precision of dosimetric evaluations, as stated by Kuprat et al. (2025), and account for diseased lungs (Oakes, 2024).

Computational Fluid and Particle Dynamics (CFPD) simulations have emerged as a viable strategy for generating subject-specific PDPs based on 3D reconstructions from CT imaging for both the TB and ET regions (Arsalanloo et al., 2022; Bartol, Graffigna Palomba et al., 2024; Feng et al., 2018; Inthavong et al., 2009; Koullapis et al., 2015; Longest et al., 2008; Zhang et al., 2022). These simulations resolve the complex airflow patterns and particle dynamics within anatomically realistic airway geometries, providing information about particle deposition that is unattainable with other models.

Moreover, in radioactive internal dosimetry, detailed radiation exposure assessments depend on understanding the exact deposition locations and quantities of radioactive particles within the lungs (Talaat et al., 2021). CFPD simulations enable detailed mapping of these deposition patterns, leading to more precise evaluations of absorbed doses. This precision is important for informing radiation protection strategies and conducting risk assessments, particularly when inhaling aerosolized radionuclide particles.

For drug delivery and inhalation therapies, particularly in treating respiratory conditions like asthma and Chronic Obstructive Pulmonary Disease (COPD), the efficacy of aerosolized medications is highly dependent on their ability to reach specific regions within the lungs (Longest & Holbrook, 2012; Rahimi-Gorji et al., 2022). CFPD simulations enable optimization of inhaler designs and dosing strategies by predicting how medications disperse and deposit within subject-specific airway geometries. This personalized approach can enhance treatment effectiveness, reduce side effects, and improve overall patient outcomes (Si & Xi, 2022; Spasov et al., 2022), but is still time-consuming and requires experts in multiple areas.

On the other hand, environmental health impact studies have also significantly benefited from CFPD simulations. Air pollutants, such as smog and fine particulate matter (PM2.5), pose serious health risks when inhaled (Zhang et al., 2006; Zhao et al., 2019). By simulating the deposition of these pollutants within the respiratory tract, investigators can better characterize exposure risks and develop strategies to mitigate adverse health effects. This is particularly important for vulnerable populations, including occupational workers exposed to hazardous environments, the general public in areas with poor air quality, and frontline responders during environmental disasters.

Despite the clear advantages of CFPD simulations, their widespread application has been limited by high computational cost and the labor-intensive process of preparing the simulation geometries. Performing individualized large-scale population simulations is complex and resource-intensive, as highlighted by Lin et al. (2021). Traditional methods for preparing computational domains involve labor-intensive processes such as manual segmentation of airway geometries from CT scans and manual capping of airway outlets and inlets to create watertight models suitable for simulation. These steps are time-consuming and introduce the potential for human error, limiting the scalability and practicality of CFPD simulations in clinical and research settings.

While CFPD has been used over the past few decades to model particle deposition in the human airways (Katz et al., 1999; Kleinstreuer & Zhang, 2010; Kleinstreuer et al., 2008), no fully automated preprocessing pipeline has been developed to date. In response to this need, this study introduces an automated workflow that reduces upfront time and resource demands required to define a computational domain for subject-specific cases. By utilizing individual-specific CT scans, this research employed advanced and traditional computer vision algorithms to generate the HRT's high-fidelity three-dimensional (3D) anatomical geometries, both from ET and TB regions. This approach addresses current limitations by automating time-consuming pre-processing steps, This approach addresses current limitations by automating time-consuming pre-processing steps, reducing manual intervention, and lowering the potential for human error.

**Fig. 1.**
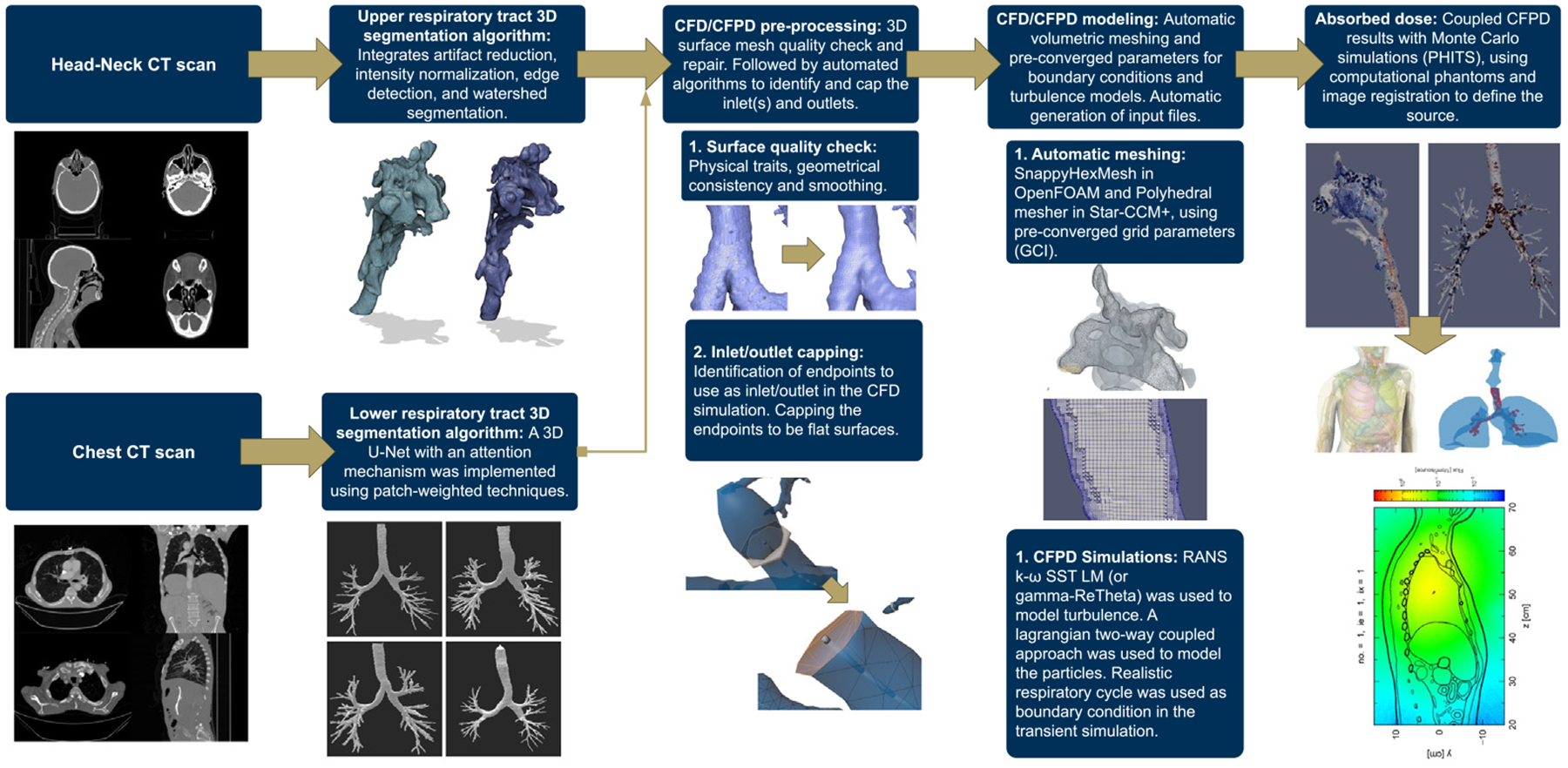
Technical summary of the proposed automated workflow. Starting from the CT scans, human respiratory tract segmentation, preparation of the geometry for a CFD/CFPD simulation, automatization of the meshing and input files, and Monte Carlo simulations for absorbed dose in the organs using computational phantoms.

The workflow also incorporated algorithms that automated the setup of CFPD simulations by generating the input files for open-source (OpenFOAM Weller et al., 1998) and licensed (Star-CCM+ Siemens Digital Industries Software, 2021) finite volume CFD/CFPD solvers. The generated PDPs provided detailed representations of particle behavior within the respiratory tract. For scenarios involving radioactive exposure, the generated PDPs can be integrated with Monte Carlo radiation transport codes to characterize the distribution of absorbed doses within the lungs. A technical graphical summary with the automated workflow presented in this manuscript can be found in Fig. 1. While MPPD and ICRP dosimetric models remain of advantage for population-level assessments and for computing deposition in the pulmonary region, the proposed workflow provides a complementary tool for applications requiring individualized resolution, such as targeted aerosol therapies, patient-specific radiation risk evaluation, and exposure analysis in vulnerable populations.

In summary, this work achieved a more precise and subject-specific assessment by introducing a scalable and efficient methodology for rapidly generating detailed PDPs within both the upper and lower regions of the HRT. While the specific time to obtain a CFD-ready 3D reconstructed model of the TB or ET airways may vary depending on multiple factors such as CT scan resolution, computational resources available, and level of metal artifacts if there are metal implants, the full pipeline typically completed in less than one hour for the most basic configuration tested, using 8 Dual Intel Xeon Gold 6226 CPUs 2.7 GHz and a NVIDIA V100 32 gb GPU for the inference of the deep learning models. This advancement has the potential to improve radiation protection models by enabling more anatomically precise airflow and deposition predictions, optimizing therapeutic interventions for respiratory diseases by supporting individualized treatment planning, and advancing knowledge of environmental health risks by providing high-resolution simulations of particulate transport and deposition in the respiratory tract.

To improve clarity and flow, this study presents the subject-specific CFD/CFPD workflow as a unified section (see Section 2) rather than separating Methods and Results. Each Subsection describes a distinct stage of the workflow, with the corresponding methodology and results presented in an integrated manner. This structure is intended to support readers who are primarily interested in specific components of the pipeline and to streamline the presentation of technical detail alongside computational outcomes.

## 2. Automated subject-specific CFD/CFPD workflow

### 2.1. Geometry reconstruction

The first step of the workflow for subject-specific studies is the reconstruction of a 3D geometry of the ET and TB regions from CT scans. The choice of imaging modality is constrained primarily by spatial resolution. While MRI has been explored for TB segmentation, its limited resolution restricts reliable identification of bronchial branches beyond the fourth generation (G4), as described by Biederer (2023). Nevertheless, for the upper part of the respiratory tract, MRI may still be suitable for reconstructing a 3D model of the nasal cavity. However, resolving the fine anatomical detail of the nasal passages remains a challenge as noted by Xiong et al. (2015).

Given these challenges, one of the main limitations of subject-specific aerosol dosimetry studies is generating a CFD-compatible geometry of a complete individual HRT. Preparing such a geometry requires that the surface mesh meet specific criteria for computational accuracy, numerical stability, and still preserve anatomical fidelity. First, the surface must be watertight (i.e. a closed, orientable 2-manifold geometry), meaning there should be no gaps or holes between adjacent elements in the geometry. Any discontinuities in the surface can result in leakage or invalid boundary conditions during the meshing and CFD simulation steps. Second, the orientation of surface normals (i.e., the normal vectors that define the direction of each surface element) must be positive and consistent. For CFD, the surface elements' outward-pointing normals define the computational domain's outside and inside. Inconsistent or inward-facing normals can lead to errors in flow directionality and volume definition during the meshing process. Moreover, the surface mesh elements (quads or triangles) must be wind-consistent. This means that the order of the nodes defining each triangular or quadrilateral surface element must follow a consistent orientation, such as all being defined in a clockwise or counterclockwise direction. Third, elements must avoid non-manifold edges and vertices. A manifold surface mesh ensures that every edge is shared by exactly two faces, ensuring the topological consistency needed for the volumetric meshing algorithms to generate valid 3D elements. The last requirement is that the geometry must have non-negative volumes and avoid degenerate or inverted elements. The surface mesh must avoid folded or self-intersecting elements, which indicate geometrical flaws and can compromise numerical calculations and CFD solvers.

In addition to geometric validity, element quality strongly influences CFD performance. High-skewness elements lead to poor volumetric meshing, resulting in numerical instability because such elements distort the local flow field representation. Surface smoothing and optimization techniques were applied to address such issues, ensuring that elements closely represent the underlying geometry without introducing artificial distortion. This is explained in detail in Section 2.4, where different smoothing techniques were compared to avoid blocky reconstructions, reduce low-quality elements, and preserve the anatomical representation of the airways.

The final step specific to CFD simulations involves identifying, defining, and preparing the geometry's inlets, outlets, and walls. These boundaries are necessary for setting boundary conditions (e.g., velocity, pressure, mass flow rate, or turbulence parameters) in CFD simulations. Inlet and outlet planes should have flat surface, be orthogonal to the expected flow direction whenever feasible, and be positioned sufficiently far from critical flow regions to avoid introducing numerical artifacts.

Another challenge encountered during the reconstruction of the HRT was that, to date, all available segmentation algorithms for this purpose have been developed exclusively for either the ET region (Cercos-Pita et al., 2018; Huang et al., 2016, 2019; Keustermans et al., 2019; Laura et al., 2019) or the TB region (Garcia-Uceda et al., 2021; Graham et al., 2008; Irving et al., 2009; Kitasaka et al., 2003; Rizi et al., 2013; Wang et al., 2022). This constraint arises primarily because CT scans are usually acquired as head or head-neck CT scans, covering the nasal cavity, oral cavity, and trachea; or, alternatively, the CT acquisition covers only the chest, making it suitable for segmenting the trachea and different generations of bronchi. Therefore, the TB and ET regions of the HRT must be analyzed separately, or a manual blending of such 3D models must be conducted beforehand.

Manual blending between the TB and ET region is a non-trivial task, requiring careful alignment and merging of models from different CT acquisitions to ensure a physically consistent transition between the different regions. Techniques like registration, surface smoothing, and interpolation are needed to connect the two parts without introducing artificial geometry artifacts, while maintaining anatomically realistic features. While different methodologies could be applied to blend two surfaces, Appendix provides a reference for the authors' recommended approach to this step.

The following subsections describe methods for reconstructing the upper and lower HRT from CT scans. Accordingly, appropriate steps were taken to ensure compliance with all the quality constraints outlined above. The workflow was organized to be modular and highly adaptable, meaning that different parts are independent and do not rely on each other as long as the input requirements for each step are met. For instance, the methodology to ensure that the 3D surface mesh is physically consistent and suitable for a CFD simulation does not rely on the segmentation algorithm used, provided that the input surface is a 3D stereolithography (STL) format file.

### 2.2. Extrathoracic region algorithm

#### Methods

**Fig. 2.**
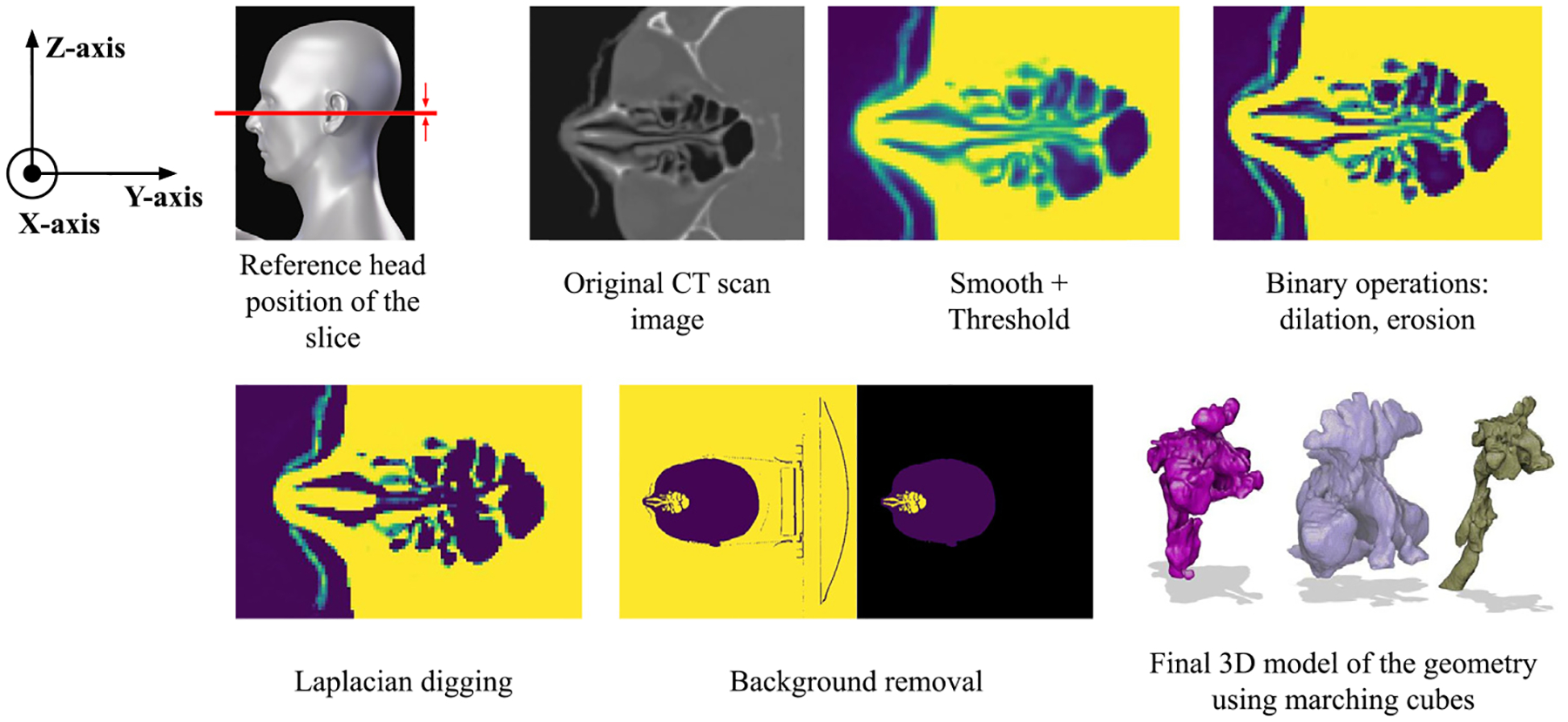
Steps of the reconstruction algorithm for the ET region. *Note:* The effect of Laplacian digging is exaggerated for visualization purposes.

An enhanced version of the segmentation algorithm developed by Cercos-Pita et al. (2018) was implemented to reconstruct the extrathoracic human respiratory tract, capturing structures such as the trachea, pharynx, sinuses, oral cavity, and nasal passages. The algorithm was modified to work with scans having a field of view (FOV) that exceeds the dimensions of the human head in the transaxial (Z-axis, detailed in Fig. 2) direction, and to include the neck as an option, a feature not supported in the original implementation. Additionally, the algorithm was further extended to capture the oral cavity in addition to the nasal cavity, while discarding disconnected or small-volume bodies. For example, small isolated volumes such as air pockets in the mastoid air cells or portions of the paranasal sinuses may be segmented as separate air regions due to partial volume effects or limited resolution of the CT scan. Similarly, nasal sinuses such as the sphenoid or ethmoid may be excluded if their ostia (connecting channels) fall below the threshold resolution of the CT scan (typically around 0.6 mm), causing them to appear as isolated cavities.

While the implementation details of the original concept can be found in the work by Cercos-Pita et al. (2018), the basic steps, modifications, and updates to the original algorithm are discussed herein. To load the CT scan imaging data, the DICOM format was chosen as the standard and most common format for handling medical imaging data (Mustra et al., 2008). Instead of relying on multiple frameworks such as GDCM (Malaterre, 2015a; Moraes, 2020) and VTK-GDCM (Malaterre, 2015b), the data manipulation task was restructured to rely solely on the PyDICOM framework from Mason et al. (2024) combined with the Visualization Toolkit (VTK) by Schroeder et al. (2006). The PyDICOM and VTK frameworks provided a more robust interface for Python, which is actively maintained and does not depend on third-party wrappers for the C code part.

The subsequent step in the algorithm involved enhancing CT image quality through the application of metal artifact reduction if there are dental fillings or implants and smoothing techniques. Metal artifact reduction was performed using a traditional inpainting-based method in the sinogram space, with metal dental fillings or implants being identified by applying intensity thresholding. A smoothing filter was applied to remove high-frequency signals (i.e., noise) while preserving edge features. The median filter, originally developed by Huang et al. (1979), was found to offer the most effective trade-off between noise reduction and edge preservation. Based on empirical tests using synthetic phantoms, median filtering with a 3 × 3 × 3 voxel size kernel reduced high-frequency noise by approximately 35%–50%, quantified by using power spectral density analysis in the region of interest. Regarding spatial resolution, it maintained voxel edge fidelity, measured by the full-width at half-maximum (FWHM) of sharp transitions (e.g., air–tissue interfaces), which did not significantly increase.

Once the CT scan was smoothed, the next step, consistent with the original implementation workflow, was to apply windowing in the data dynamic range. In CT imaging, this range is expressed as the Hounsfield Units (HU) values, a scale used to represent the relative density of tissues based on their X-ray photon attenuation, with air set at −1000 HU, water at 0 HU, and denser materials like bone having positive values. After the windowing step, voxel intensities were linearly normalized between 0 and 1, as summarized in Eq. (1).

(1) $$I_{i}^{*}=\left\{\begin{array}{ll} 0 & \text{if}\;I_{i}\leq I_{0,}\\ 1 & \text{if}\;I_{i}\geq I_{1},\\ \frac{I_{i}-I_{0}}{I_{1}-I_{0}} & \text{otherwise.} \end{array}\right.$$

In this work, the value for $I_{0}$ was set by default at −900 HU, meaning that anything below this value was considered air. In contrast, for $I_{1}$, the default value was set to −115 HU (fat threshold), and everything above this was classified as tissue. Once the mask was created, a Canny edge detection algorithm (Canny, 1986) was applied in the NASAL-Geom pipeline.

The Canny edge detection algorithm was applied to effectively identify edges by detecting local maxima in the intensity gradient while accounting for gradient direction. This process involved three steps: (1) detecting edges using the Canny algorithm and storing them in a mask, (2) applying morphological operations (binary dilation, hole filling, and erosion) to refine the edges and select enclosed voxels, and (3) amplifying the radiosity range of voxels in the mask by raising their values to the third power to highlight relevant features.

To further improve cavity segmentation, a filter based on the Laplacian edge detector was employed to recover larger cavities. While the Canny detector was robust against noise, the Laplacian detector offered two further advantages: (1) assigning positive values to the darker side of edges, which enabled enhancement of large holes by subtracting the Laplacian from the original image, and (2) offering better suitability for capturing three-dimensional features, such as thin, flat surfaces. The Laplacian edge detection can be computed as in Eq. (2).

(2) $$\nabla^{2}I=\frac{\partial^{2}I}{\partial x^{2}}+\frac{\partial^{2}I}{\partial y^{2}}+\frac{\partial^{2}I}{\partial z^{2}}$$

In Eq. (2), I represents the image intensity in HU values, $\frac{\partial^{2}I}{\partial x^{2}}$, $\frac{\partial^{2}I}{\partial y^{2}}$, and $\frac{\partial^{2}I}{\partial z^{2}}$ denote the second partial derivatives of the image I with respect to the x, y, and z coordinates, respectively. Here, x and y refer to the in-plane axes of the CT scan, while z corresponds to the through-plane direction. Following this step, a 3D watershed algorithm was employed to recognize air pockets, but further refinement was required due to the nostrils being connected to the background air. A 2D watershed algorithm was applied to create axial and coronal masks to recognize the background, as specified by Cercos-Pita et al. (2018). The original algorithm proposed the exclusion of isolated bodies smaller than 15 cm^3^ when more than three bodies were found per slice. However, this refinement was found to potentially eliminate desired structures, such as the oral cavity and sinuses. Therefore, the segmentation function was refined to include the nostrils, oral cavity, and sinuses. A criterion for identifying the oral cavity based on the volume range of (3–9.66) cm^3^ as reported by Rana et al. (2020) was added. The lower limit for the oral cavity volume was determined through iterative trials on 40 subjects, and the upper limit represents the mean volume plus one standard deviation derived from empirical data. The dataset used for this work was obtained from the Head-Neck Cetuximab database (Ang et al., 2014; Bosch et al., 2015), which contains CT scans from 111 CT patients age 42 to 76 years old, 15% of whom were female.

The generated surface in its raw form is not suitable for CFD simulation, and the geometry preprocessing stage (Section 2.4) provides a detailed algorithm to achieve the required quality. The pseudo-algorithm for the reconstruction of the ET region is presented in Algorithm 1, and a visual representation of the steps is shown in Fig. 2.

**Algorithm 1:** Upper Respiratory Tract Segmentation Algorithm

1:	**Input:** DICOM CT scan of the upper respiratory tract	
2:	**Output:** Segmented 3D surface geometry	
3:	**Step 1: Load and Preprocess Data**	
4:	I←LoadDICOM(CTscan)	// Read DICOM images using PyDICOM and VTK
5:	I←MetalArtifactReduction(I)	// Reduce metal artifacts in sinogram space
6:	I←MedianFilter(I)	// Remove noise while preserving edges
7:	**Step 2: Windowing and Normalization**	
8:	**for all** voxels $I_{i}$ in I **do**	
9:	$I_{i}^{*}\leftarrow \text{NormalizeIntensity}(I_{i},I_{0},I_{1})$	
10:	**end for**	
11:	**Step 3: Edge Detection and Refinement**	
12:	E←CannyEdgeDetection(I*)	// Detect edges
13:	E←MorphologicalOperations(E,dilation,holefilling,erosion)	// Refine edges
14:	**Step 4: Laplacian-Based Enhancement**	
15:	L←Laplacian(I*)	// Curvature-based enhancement
16:	$I_{\text{enhanced}}\leftarrow I*-L$	
17:	**Step 5: Nasal and Oral Cavity Segmentation**	
18:	$V_{\text{air}}\leftarrow \text{3DWatershed}(I_{\text{enhanced}})$	// Identify air regions
19:	**for all** slices $I_{i}^{\text{coronal}}$, $I_{i}^{\text{sagittal}}$ in $I_{\text{enhanced}}$ **do**	
20:	$V_{\text{air},\;i}^{\text{refined}}\leftarrow \text{2DWatershed}(I_{i}^{\text{coronal}})\cup \text{2DWatershed}(I_{i}^{\text{sagittal}})$	
21:	**end for**// Identify air from background	
22:	$S_{\text{air}}^{\text{refined}}\leftarrow \text{SplitVolumes}(V_{\text{air}}^{\text{refined}})$	// Split air regions
23:	$V_{\text{nasal}}\leftarrow \text{GetLargestBodies}(S_{\text{air}}^{\text{refined}})$	// Get nasal region
24:	**for all** volumes $V_{\text{air},\;i}^{\text{refined}}$ in $S_{\text{air}}^{\text{refined}}$ **do**	
25:	**if** $V_{\text{air},\;i}^{\text{refined}}\in (3,9.66){\text{cm}}^{3}$ **then**	
26:	$V_{\text{nasal}}\cup V_{\text{air},\;i}^{\text{refined}}$	// Merge oral cavity and sinuses
27:	$V_{\text{nasal}}\leftarrow \text{RefineBodies}(V_{\text{nasal}})$	// Connect bodies and refine
28:	**end if**	
29:	**end for**	
30:	**Step 6: Generate 3D Surface Mesh**	
31:	$M\leftarrow \text{MarchingCubes}(V_{\text{nasal}})$	// Extract 3D surface using Marching Cubes
32:	ExportMesh(M)	// Save surface mesh as a STL
33:	**End**	

#### Results

A total of 111 CT scans were segmented from different publicly available datasets, as detailed in Section 2.8. From the dataset of 111 ET segmentations, some including nasal and oral cavities while others only nasal cavity, 40 geometries were manually analyzed for leaks, holes, or any other non-physical features in the segmentation that resulted from algorithmic error rather than CT scan artifacts. Of the 40 segmented upper HRTs, seven were discarded due to non-physical features unrelated to CT scan quality. Based on this inspection, the algorithm achieved a success rate of 82.5%.

**Fig. 3.**
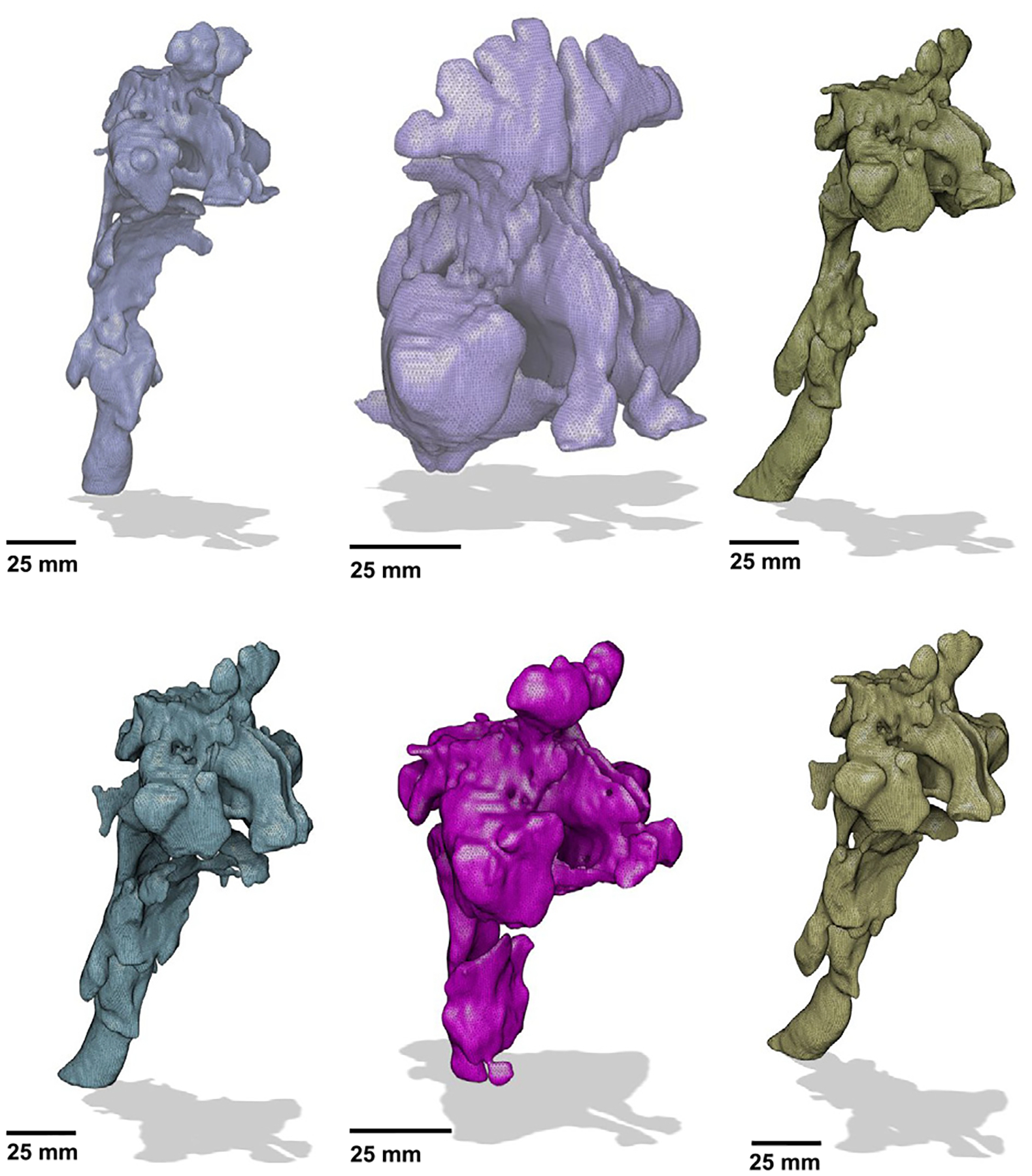
Results from the ET region segmentation algorithm applied to different head and head–neck CT scans. The paranasal sinuses are captured, as well as the oral cavity when connected. *Note:* The scale provides an approximate reference for dimensions and is not intended for precise measurement.

Fig. 3 presents representative examples of segmented geometries, highlighting anatomical variations across different regions of the nasal cavity. Among these, the pharynx region exhibited the greatest inter-subject variability, with both the nasopharynx and oropharynx varying in size and shape. This variability was likely influenced by the patient’s breathing state during the CT acquisition. Some subjects may have demonstrated mixed breathing (oral and nasal), while others exhibited exclusive nasal breathing, leading to greater continuity in the oropharyngeal region. In all the cases, the frontal and maxillary sinuses were identified and segmented.

#### 2.2.1. Limitations

Several limitations should be acknowledged for the ET respiratory tract segmentation algorithm, despite the robustness of the proposed approach. Firstly, anatomical variability between different inhalation rates and respiratory states cannot be captured in a CT scan, given that the subject is always a resting condition, or in a inhalation or exhalation hold state. Therefore, the shape and dimensions of structures such as the larynx and pharynx can differ significantly depending on the breathing rate and if the CT was captured during inhalation or exhalation hold (Schwab et al., 1993). These changes may lead to geometrical discrepancies between the acquired geometry from the CT scan and the actual anatomy during the simulated airflow conditions, potentially affecting CFD/CFPD predictions.

**Fig. 4.**
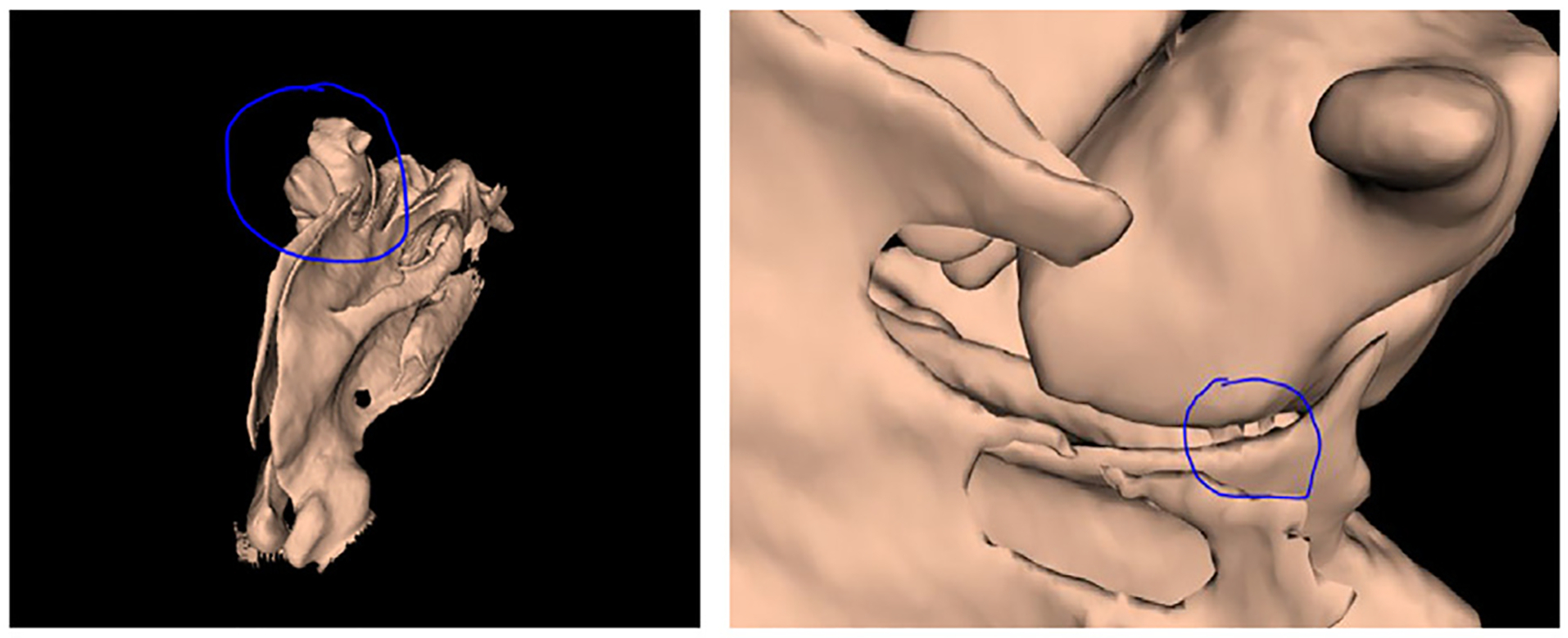
Example of non-physical traits from the nasal cavity segmentation that were found from manually inspecting different segmentations. In this example, leakage was observed in the nasal passages to the sinuses in a region that should not be connected.

Additionally, small structures, or structures partially occluded in the CT scans due to respiratory or motion artifacts, may still be missed or improperly reconstructed, as illustrated in Fig. 4. The algorithm effectiveness is indeed influenced by the selected thresholding and filtering parameters, which, although optimized through extensive testing, may require manual adjustment for cases with extreme anatomical variation. Finally, residual artifacts or incomplete segmentation, particularly around regions with metal implants or dental fillings, might occasionally require manual intervention, thus limiting full automation of the process for a subset of clinical cases.

### 2.3. Tracheobronchial region algorithm

#### Methods

For the segmentation of the TB region, including the trachea (G0) up to the bronchioles, multiple algorithms have been developed over time, beginning with morphology-based approaches (Graham et al., 2010; Irving et al., 2009; Kitasaka et al., 2003; Lai et al., 2009; Lo et al., 2010; Reynisson et al., 2015; Rizi et al., 2013), which utilized techniques such as thresholding, leakage suppression, binary operations, and anatomical knowledge of the airways. While these algorithms accurately segment the larger structures of the airway tree — such as the trachea and main bronchi — performance typically declines beyond the fifth bronchial generation (G5) (Reynisson et al., 2015; Rizi et al., 2013).

To address this limitation, recent work has focused on deep learning (DL)-based algorithms (Garcia-Uceda et al., 2021; Nadeem et al., 2021; Qin et al., 2019; Wang et al., 2022), which have significantly advanced TB segmentation, especially in finer bronchi structures. These methods have made use of the publicly available EXACT'09 dataset (Lo et al., 2012), which includes 46 CT scans with expert-labeled airway structures for training and validation. One of the primary advantages of DL-based approaches is the ability to reconstruct more distal branches of the airway tree, with some models—such as that by Wang et al. (2022)—achieving segmentation down to the 12th bronchial generation (G12), depending on CT image quality.

**Fig. 5.**
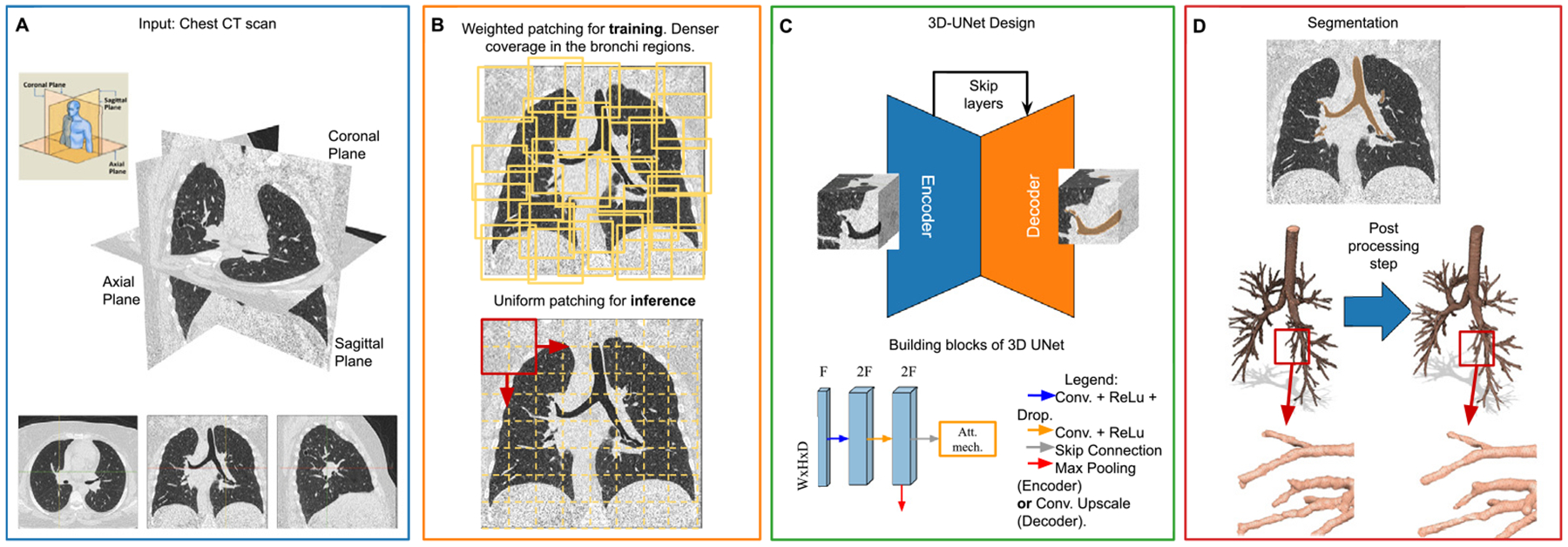
(A) A volumetric CT scan in the format of a DICOM file served as input data for the neural network. For training an extra channel with the labeled airways must be provided. (B) Patching used for training (up) and inference (bottom). A weighted approach was used for training to better capture finer structures such as lower generations of bronchi, where there are fewer voxels per input volume. A structured mapping approach was used for inference. (C) A visual representation of the general 3D-UNet design is provided on the figure on top, emphasizing the 3D input volume representation and the label as the output. In the bottom figure, a schematic representation of the building blocks of the 3D UNet are shown, and W, H, D, and F are referring to width, height, deep, and features respectively. (D) Displays an example of the output by the neural network on top, the output of the marching cubes algorithm in the middle left, and a post-processed geometry on the middle-right. At the bottom of (D) details of the post-processing step are displayed.

In the present study, a 3D U-Net architecture based on the Navi-Airway framework (Wang et al., 2022) was employed to segment the lower respiratory tract. While a summary of the network architecture and training process is given below, the implementation details can be seen in the original work by Wang et al. (2022). The backbone model was a 3D U-Net neural network, an encoder–decoder with an integrated attention mechanism, as depicted in Fig. 5C. This attention mechanism was designed to improve airway representation by incorporating three types of image-level features. First, global contextual information was aggregated via adaptive average pooling, which reduced each feature map to a single representative statistic. Second, channel-wise dependencies were learned through a fully connected network, which generated scaling coefficients for each channel. Third, these coefficients were used to reweight the features across channels, enhancing spatial and contextual relevance. The attention mechanism was applied in parallel with dilated convolution operations in both encoder and decoder blocks. The implementation of the 3D U-Net can be seen in Algorithm 2. The loss function used for training combined three components to support accurate segmentation across multiple bronchial generations. The first term applied a weighted dice coefficient between the ground truth and the prediction, calculated specifically on the skeletonized airway structure (i.e., a centerline-like representation). This skeleton-based term enhanced topological fidelity and improved the detection of smaller bronchi. The second and third components targeted larger anatomical structures, such as the trachea and main bronchi. For these, the loss function was proposed as the sum of two dice coefficients at a voxel level: one to penalize misclassified voxels in the background and another to penalize misclassified voxels in the airway structure using a weighted dice loss coefficient.

To make the neural network prediction independent of the input size of the CT scan, the input volume was first cropped into smaller volumes of 128 × 128 × 64 voxels (the size in mm depend on the voxel size of the CT scan), where the last dimension is the transaxial dimension (Z-axis, detailed in Fig. 2). Furthermore, since the lower generations of bronchi (G3 and beyond) contain significantly fewer voxels than the upper generations (trachea, G1, and G2), an iterative training shuffle and weighted patching were applied to pass through the network multiple times those sub-volumes containing distal airway generations, represented by the number of mask voxels in the input volume. The input volume dimensions may also be larger or smaller, depending on the GPU memory available to fit the model. In this study, training was performed using a single NVIDIA A100 Tensor Core with 40 GB of memory. Data augmentation was performed by applying rigid transformations (small random rotations up to 10 degrees, axis flipping, and random scaling within the range of (0.75–1.25)); these transformation parameters were adopted from Garcia-Uceda et al. (2021). After the training process, each patch generated a patch-wise airway probability map.

The post-processing method proposed by Garcia-Uceda et al. (2021) was applied for the final reconstruction step. The process of constructing a final 3D model from the patch outputs by the neural network was as follows:

1. **Probability patch volume generation:** Each airway probability patch volume generated was reassembled by following the reverse process of generating the patches. Any overlap between volumes was weighted by multiplying them together, returning a new airway probability map that contains all the candidate branches and segments.
2. **Volume patch constraining:** To eliminate any non-physical prediction, the airway mask was constrained to the lungs using a predefined lung mask obtained by simply thresholding between [−1000, −600] HU. The lung mask needs to be fast, and anatomical precision was not prevalent in this case since the algorithm does not include any of the alveolar or bronchiolar region, which, if that would be the case, it would require a more detailed lung mask.
3. **Binary volumetric patch:** To create a binary mask of the airway map, a probability threshold of 0.5 was applied to convert the mask to a binary segmentation.
4. **Final volume generation:** To satisfy morphological continuity and eliminate minor artifacts that may have appeared from neural network output, the final airway tree was extracted by selecting the largest 26-nearest neighbor connected components (voxels) in the structured cubic grid, representing the most likely airway structure.

A visual summary of the steps for obtaining the TB region airways from CT scans can be seen in Fig. 5.

**Algorithm 2:** 3D U-Net Backbone Architecture

**Require:** Input volumetric image $X\in {\mathbb{R}}^{C\times H\times W\times D}$, where C is the number of channels, and H, W, D are the height, width, and depth respectively.	
**Ensure:** Segmentation output $Y\in {\mathbb{R}}^{N\times H\times W\times D}$ where N is the number of classes.	
1:	**// Encoder Path: Each pooling halves the spatial dimensions**
2:	$E_{1}\leftarrow \text{EncoderModule}(X)$ {Encoder 1: Channels: C → 16 → 32; **No pooling;** Output dims: 32×H×W×D}
3:	$E_{2}\leftarrow \text{EncoderModule}(E_{1})$ {Encoder 2: Channels: 32 → 32 → 64; **With pooling: $\text{dims}\to 64\times \frac{H}{2}\times \frac{W}{2}\times \frac{D}{2}$**}
4:	$E_{3}\leftarrow \text{EncoderModule}(E_{2})$ {Encoder 3: Channels: 64 → 64 → 128; **With pooling: $\text{dims}\to 128\times \frac{H}{4}\times \frac{W}{4}\times \frac{D}{4}$**}
5:	$E_{4}\leftarrow \text{EncoderModule}(E_{3})$ {Encoder 4: Channels: 128 → 128 → 256; **With pooling: $\text{dims}\to 256\times \frac{H}{8}\times \frac{W}{8}\times \frac{D}{8}$**}
6:	**// Decoder Path: Each upsampling doubles the spatial dimensions**
7:	$D_{1}\leftarrow \text{DecoderModule}(E_{4},E_{3})$ {Decoder 1: Upsample: 256 → 256 ($\text{dims}:\frac{H}{8}\to \frac{H}{4}$, etc.), concat with $E_{3}$ (128 channels), output: 128 channels; $\text{dims}:128\times \frac{H}{4}\times \frac{W}{4}\times \frac{D}{4}$}
8:	$D_{2}\leftarrow \text{DecoderModule}(D_{1},E_{2})$ {Decoder 2: Upsample: 128 → 128 ($\text{dims}:\frac{H}{4}\to \frac{H}{2}$, etc.), concat with $E_{2}$ (64 channels), output: 64 channels; $\text{dims}:64\times \frac{H}{2}\times \frac{W}{2}\times \frac{D}{2}$}
9:	$D_{3}\leftarrow \text{DecoderModule}(D_{2},E_{1})$ {Decoder 3: Upsample: 64 → 64 ($\text{dims}:\frac{H}{2}\to H$, etc.), concat with $E_{1}$ (32 channels), output: 32 channels; dims:32×H×W×D}
10:	**// Final Segmentation Layer**
11:	$Y\leftarrow \text{FinalConv}(D_{3})$ {3D convolution: 32→N, kernel size 3 × 3 × 3, padding = 1; dims:N×H×W×D}
12:	Y←Softmax(Y) {Apply softmax along the channel dimension}
13:	**return Y**

#### Results

**Fig. 6.**
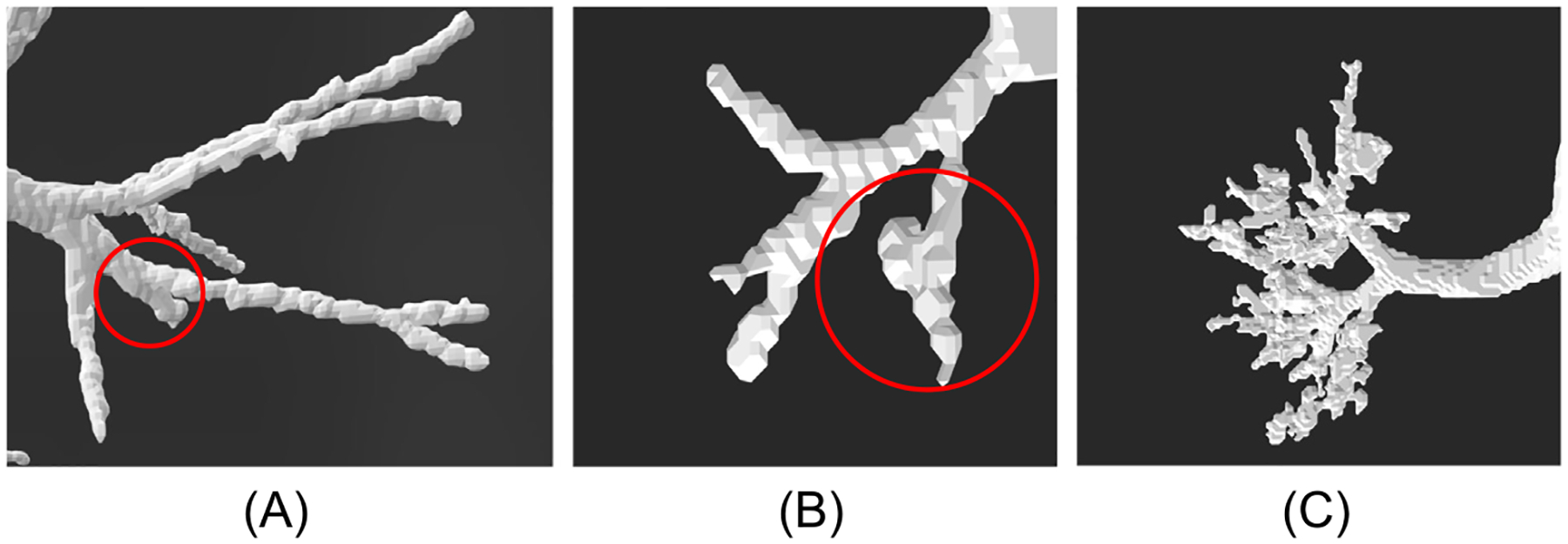
Examples of the criteria applied to discard geometries after manual visual inspection. (A) Example of a non-physical bronchi, where the algorithm incorrectly tried to continue the previous generation of bronchi in a direction that did not exist in the CT scan. (B) Example of a non-physical terminal bronchi. (C) Example of an unrealistic segmentation of the tracheobronchial tree, due to motion artifacts in the lungs.

**Fig. 7.**
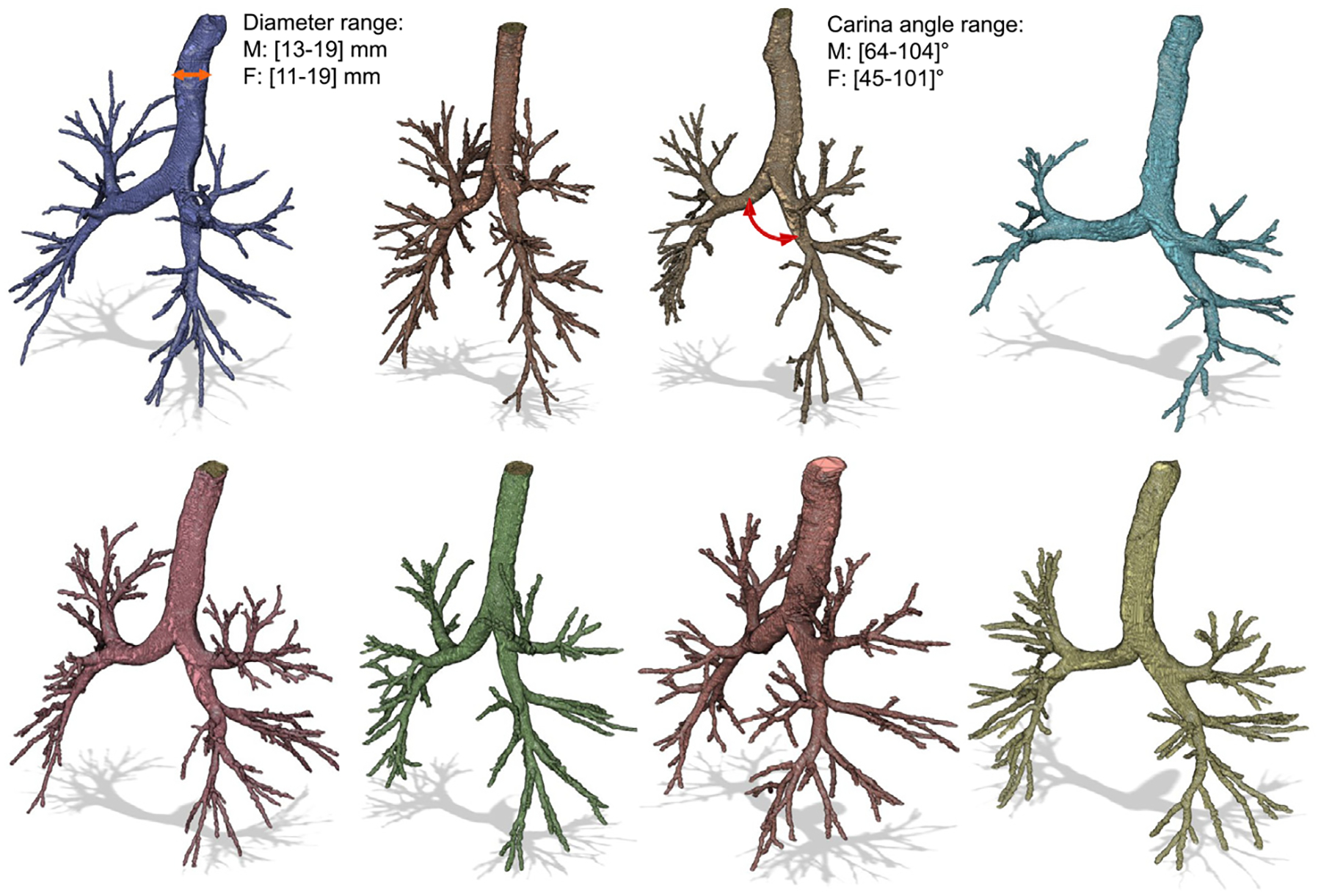
Results of the TB region airways segmentation algorithm applied to chest CT scans. The figure illustrates the substantial variability in human airway morphology across individual subjects. The geometries shown have not been post-processed to evaluate surface mesh quality, which constitutes the next step in the pipeline. The displayed ranges represent the average tracheal diameter (in millimeters) and carina ridge angle (in degrees) for the analyzed cohort, reported separately for male (M) and female (F) subjects.

The results of the modified segmentation algorithm for the TB region airways are presented in Fig. 7. A total of 412 three-dimensional lower HRT geometries were reconstructed from CT scans of subjects ranging in age from 2 to 90 years. The cohort consisted of 54% male and 46% female subjects, including 88 pediatric subjects (ages 2 to 12 years old). The modified algorithm achieved a success rate of 76%, excluding segmentation anomalies such as non-physical bronchi or unrealistic airway geometries, issues that have also been noted in prior studies (Garcia-Uceda et al., 2021; Graham et al., 2008; Shariaty et al., 2019; Wang et al., 2022). Examples of such cases are shown in Fig. 6.

Another factor affecting segmentation performance was the quality and resolution of the CT scans, which influenced the number of discernible bronchial generations captured by the algorithm. Table 1 summarizes the variability in voxel dimensions from the CT datasets used in this study. The details of the CT scan sources are provided in Section 2.8. The number of bronchi generations captured by the algorithm never fell below the 4th generation of bronchi (G4), and, at most, reached the 8th generation of bronchi (G8) based on the number of outlets identified. Although Wang et al. (2022) reported segmentation of bronchiolar structures up to G12, the datasets used in this work did not yield anatomically consistent segmentations beyond G8.

**Fig. 8.**
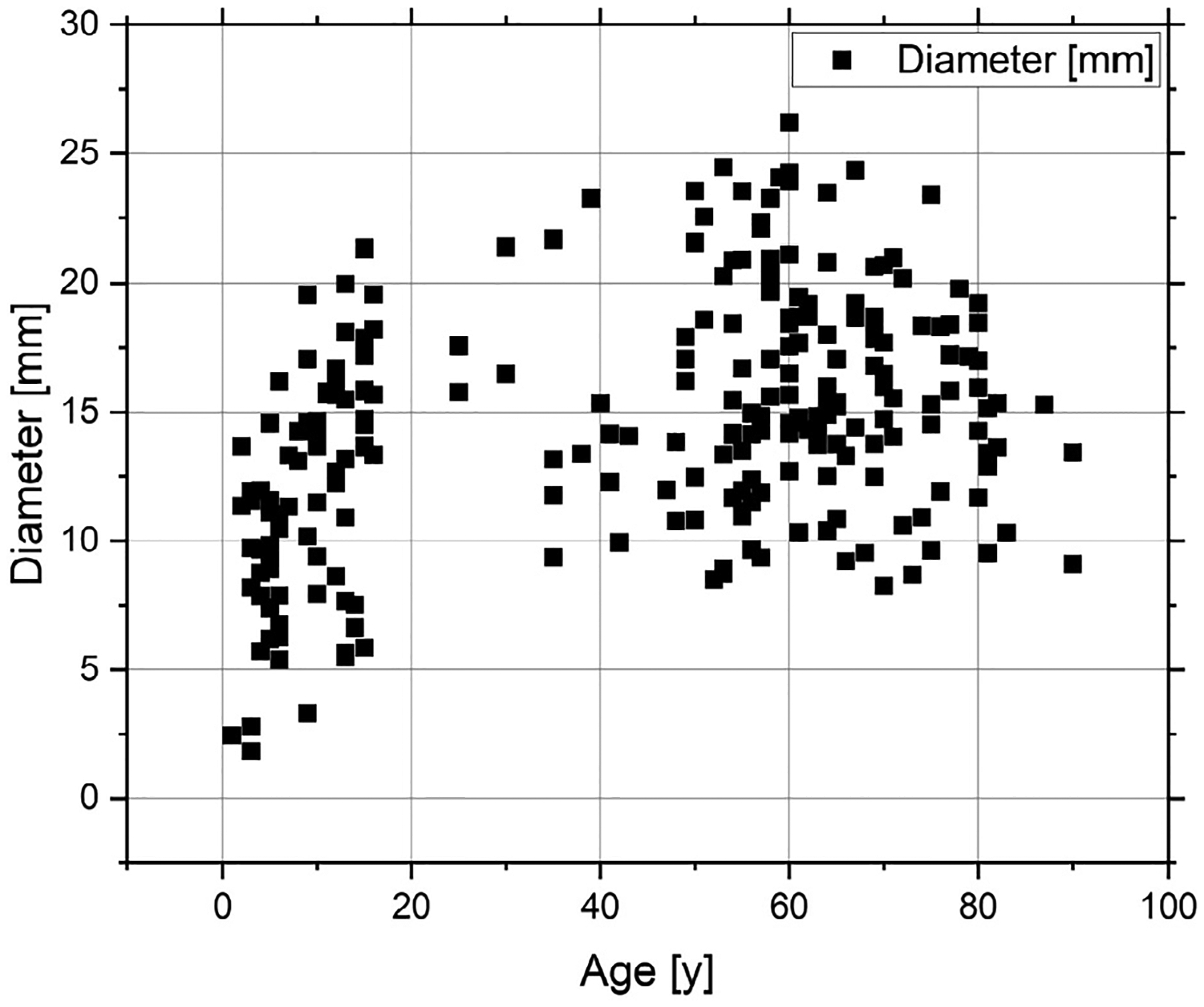
Scatter plot showing tracheal diameter (mm) of segmented airway models as a function of subject age (years). The data demonstrate a marked increase in tracheal diameter during early childhood, followed by a plateau in adolescence to adulthood. This plot demonstrates the age-dependent anatomical development of the trachea, which may be relevant for informing age-specific aerosol dosimetry.

Fig. 8 shows the variation in tracheal diameter as a function of subject age. A total of 257 airway segmentations with available age data, including both male and female subjects, were analyzed. The plot shows that tracheal diameter increases rapidly during early childhood, then stabilizes and remains within a narrow range in adult subjects.

#### 2.3.1. Limitations

The performance of the segmentation approach for the TB region is strongly dependent on the quality of the input CT scans. Respiratory and cardiac motion artifacts, which are common during CT acquisition, were not represented in the algorithm's training dataset (EXACT'09), which exclusively contains scans acquired during breath-holds at either full inspiration or expiration. Consequently, scans with motion artifacts, common in clinical 4D-CT (or cine CT mode in some vendors) acquisitions without respiratory gating, resulted in substantially reduced segmentation performance, especially in distal bronchial generations (G4 and beyond) that are more susceptible to image degradation.

In addition, physiological airway deformations across the respiratory cycle (e.g., inhalation vs. exhalation) introduce structural variability that is not related to CT image quality or segmentation error. For example, the carina ridge in a 4D-CT scan from the DIR-Laboratory dataset (Castillo et al., 2009) was displaced by approximately 5 mm between full inspiration and expiration. This suggests that airway geometries extracted from static breath-hold scans may not fully capture dynamic respiratory mechanics, which can influence subsequent CFD or CFPD simulations aimed at modeling realistic breathing patterns.

### 2.4. HRT geometry processing for CFD simulations

**Fig. 9.**
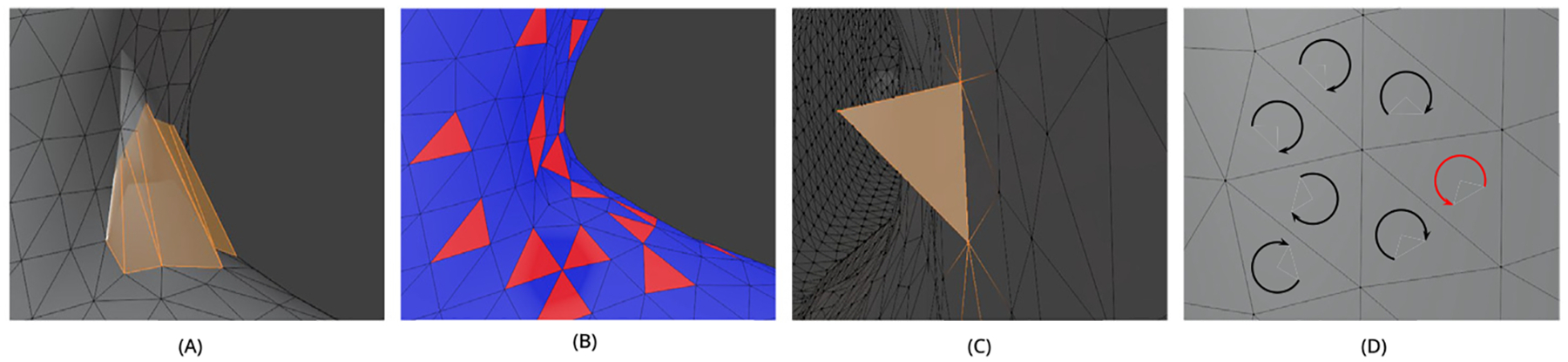
Common meshing errors from the marching cubes algorithm. (A) Self-intersecting faces are usually common in bifurcating branches with small angles. (B) Negatively oriented surface, where the normal of the face is pointing inward the volume. (C) Example of a non-manifold edge. While the definition of a non-manifold edge is complex, an acceptable definition is when an edge is shared by more than two faces or just one face (standalone face), which makes it physically impossible in the real world because the geometry does not define a clear inside and outside or volume. (D) Inconsistent winding orientation. All surface elements must have a consistent order of the vertex so that the outward-facing normals point away from the fluid domain. A polygonal mesh with consistent winding has each shared edge going in an opposite direction from the other in the pair.

Sections 2.2 and 2.3 outlined procedures used to generate volumetric segmentation of the airways in the ET and TB regions. To convert these segmentations into surface-based geometric representations, the marching cubes algorithm was applied to produce a polygonal surface mesh for each HRT region. For use in CFD or CFPD simulations, the resulting 3D surface mesh must be free of geometric artifacts and represent a physically plausible structure. Surface meshes derived directly from voxel-based segmentations often exhibit non-physical geometric artifacts, including self-intersecting faces, inward-facing (negatively oriented) normals, non-manifold edges, and inconsistently face winding. Examples of these artifacts in HRT meshes are shown in Fig. 9.

To address these geometric issues, a sequence of mesh correction procedures was applied. First, non-manifold edges were identified and removed using a targeted detection and elimination algorithm. Let ℳ=(𝒱,ℱ) be the triangular surface mesh of the HRT, where:

- $𝒱\subset R^{3}$ is the set of vertices.
- $ℱ\subset {𝒱}^{3}$ is the set of triangular faces.
- ℰ is the set of edges, where each edge is shared by at least one face.

By definition, a non-manifold edge e∈ℰ is an edge that is shared by more than two faces, as explained in Fei et al. (2013). The first step was then to identify non-manifold edges. Each edge $e_{ij}=\left(v_{i},v_{j}\right)\in ℰ$ connects two vertices $v_{i}$ and $v_{j}$. An edge-face incidence function was defined as:

(3) $$\text{count}\left(e_{ij},ℱ\right)=\left|\left\{f\in ℱ|e_{ij}\in f\right\}\right|,$$

where $\text{count}\left(e_{ij},ℱ\right)$ gives the number of faces containing edge $e_{ij}$.

The set of non-manifold edges was selected as

(4) $${ℰ}_{nm}=\left\{e_{ij}\in ℰ|\text{count}\left(e_{ij},ℱ\right)>2\right\}.$$

Faces associated with non-manifold edges were identified to remove them later. A face f∈ℱ was considered as a candidate for removal if it was composed of at least one non-manifold edge:

(5) $${ℱ}_{nm}=\left\{f\in ℱ|f\cap {ℰ}_{nm}\neq \emptyset \right\},$$

subsequently, to obtain an updated mesh ${ℳ}^{\prime }=\left({𝒱}^{\prime },{ℱ}^{\prime }\right)$, all faces associated to non-manifold edges were removed:

(6) $${ℱ}^{\prime }=ℱ\setminus {ℱ}_{nm},$$

where \ operand denotes the set difference operation and removes all elements in ${ℱ}_{nm}$ from ℱ. After removing non-manifold associated faces, all the orphaned or isolated vertices must also be removed. If a vertex was not part of any face in ${ℱ}^{\prime }$, was removed following,

(7) $${𝒱}^{\prime }=\left\{v\in 𝒱|\exists f\in {ℱ}^{\prime },v\in f\right\}.$$

To ensure that the resulting mesh ${ℳ}^{\prime }=\left({𝒱}^{\prime },{ℱ}^{\prime }\right)$ was manifold, the following condition was checked for all the remaining edges:

(8) $$\forall e\in {ℰ}^{\prime },\;1\leq \text{count}\left(e,{ℱ}^{\prime }\right)\leq 2,$$

This condition guarantees that each edge was shared by at most two faces. The resulting mesh ${ℳ}^{\prime }$ was not guaranteed to be watertight, have consistent winding orientation, or have a positive volume. Therefore, additional steps were required to enforce watertightness and correct the orientation of any misaligned faces.

Following the same notation as before, let ${ℳ}^{\prime }=\left({𝒱}^{\prime },{ℱ}^{\prime }\right)$ be a triangular mesh where ${𝒱}^{\prime }\subset R^{3}$ represents the set of vertices and ${ℱ}^{\prime }\subset {𝒱}^{3}$ represents the set of triangular faces. An algorithm was applied to detect and fill missing faces such that the resulting mesh, denoted ${ℳ}^{\prime \prime }$, became watertight. A mesh was considered watertight if each edge e∈ℰ is shared by exactly two faces. In other words, the geometry is closed and an orientable 2-manifold surface. The set of boundary edges ${ℰ}_{b}$ is defined as:

(9) $${ℰ}_{b}=\left\{e\in ℰ|\text{count}\left(e,{ℱ}^{\prime }\right)=1\right\},$$

where $\text{count}(e,{ℱ}^{\prime })$ denotes the number of occurrences of edge e in the face set ${ℱ}^{\prime }$. To efficiently group edges forming a hole, the algorithm constructed a boundary graph $𝒢=\left({𝒱}_{b}^{\prime },{ℰ}_{b}\right)$, where ${𝒱}_{b}\subset {𝒱}^{\prime }$ were the vertices associated with ${ℰ}_{b}$. The hole cycles were detected using a cycle basis extraction function from Hagberg et al. (2008), implemented in the NetworkX Python Package:

(10) ℋ=cycle_basis(𝒢).

Each hole h∈ℋ is a polygon of vertices $v_{i}$. To fill the holes, the following steps were applied:

1. If |h|=3, the hole is a missing triangle and is directly added to ℱ:
(11) ℱ←ℱ∪{h}.
2. If |h|=4, it is decomposed into two new triangles:
(12) $$ℱ\leftarrow ℱ\cup \left\{\left(v_{1},v_{2},v_{3}\right),\left(v_{3},v_{4},v_{1}\right)\right\}.$$

For larger holes (|h|>4), a constrained Delaunay triangulation method was used (Sloan, 1993), although not explicitly covered in this manuscript for larger, the scipy.spatial Python Delaunay triangulation implementation was used from Virtanen et al. (2020).

Newly added faces $f=\left(v_{a},v_{b},v_{c}\right)$ were constrained to maintain consistent winding order with adjacent faces. The normal vector n of a triangle was computed as $n=\left(v_{b}-v_{a}\right)\times \left(v_{c}-v_{a}\right)$. The correct face winding is enforced by checking edge orientation consistency, where $e_{\text{shared}}$ is the edge shared by two adjacent faces in a polygon mesh. It was defined that $e_{\text{shared}}$ is reversed when the traversing direction of the node in the edge was opposite for two adjacent faces; therefore, the face was flipped following Eq. (13),

(13) $$\text{flip}(f)\;\text{if}\;e_{\text{shared}}\;\text{is}\;\text{not}\;\text{reversed.}$$

The process was repeated through all holes h∈ℋ. Once all holes were processed:

- The new faces were added to the mesh: ${ℳ}^{\prime \prime }=\left({𝒱}^{\prime \prime },{ℱ}^{\prime \prime }\right)$.
- Vertex merging was performed, meaning duplicate vertices within a tolerance $\epsilon \leq 1\times 10^{-6}\;\text{cm}$ were merged to reduce skewness.

The resulting mesh ${ℳ}^{\prime \prime }$ was watertight, with the set ${ℰ}_{b}=\emptyset$. Since the hole-filling procedure does not guarantee that only manifold edges are added, an iterative refinement procedure was applied. At each iteration, non-manifold edges were identified and removed, and watertight check was reassessed. The process was repeated until the mesh satisfied both conditions. While a priori, an endless loop may occur and a maximum number of iterations should be set. Extensive testing across more than 400 geometries demonstrated that convergence was always achieved within three iterations.

**Fig. 10.**
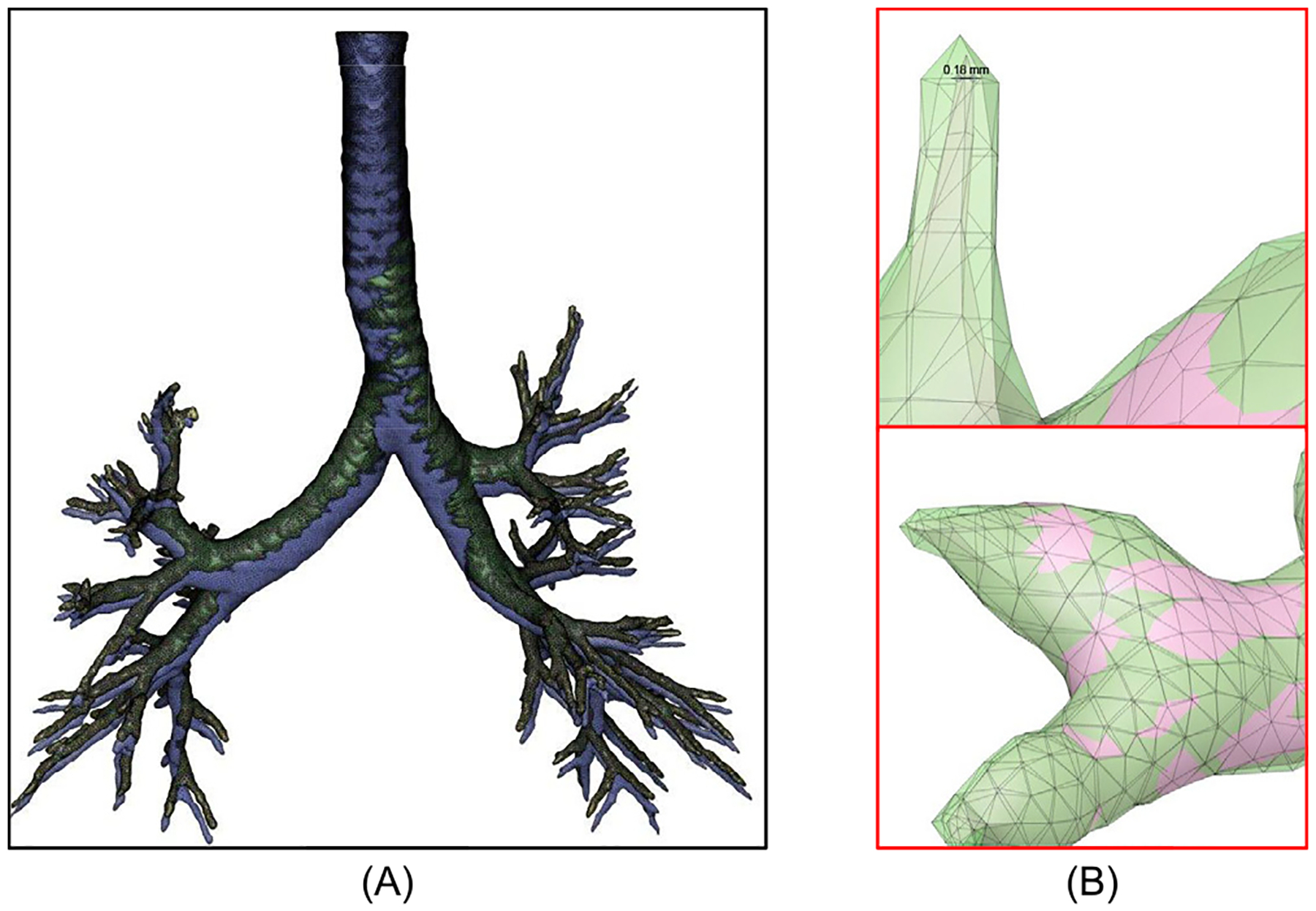
Difference in smoothing filters for polygon meshes. (A) When a Laplacian smoothing is applied, the geometry shrinks and changes its volume (green geometry); instead, when a Taubin filter is applied, the geometry keeps its volume and does not shrink (blue geometry). The original geometry is also pictured but overlaps with the blue geometry. (B) The difference when using a Laplacian smoothing filter with volume preservation led to pointy tips and shrinkage, whereas a Taubin smoothing filter (green surface) avoided this degradation in quality.

Moreover, further geometric quality constraints must be satisfied when the geometry is intended for CFD simulations, as explained in Attene et al. (2013). The surface polygon mesh of the HRT must be free of topological noise (low skewness, smooth geometry) and degenerated faces (faces with near-zero area), avoiding the common GIGO (“garbage in, garbage out”) problem in CFD. To reduce mesh skewness, a remeshing operation was performed using the Polygon Mesh Repair package from CGAL (Coeurjolly et al., 2024), implemented in C++. Subsequently, a smoothing filter was applied to the polygon mesh to refine the geometry. The Taubin filter (Taubin, 1995; Vollmer et al., 1999) was selected because it preserves volume and minimizes shrinkage, thereby retaining finer bronchial structures. This filter has also been shown to reduce sharp wall edge transitions and surface artifacts that may otherwise introduce to numerical errors during volumetric meshing or simulation. The smoothing operation modified vertex positions locally and iteratively to reduce noise while preserving fine anatomical detail. The Taubin smoothing filter has two parameters: λ, which controls shrinkage, and has a valid range of 0.0≤λ≤1.0, and a dilation parameter ν, which controls expansion, in the range of 0.0≤v≤1.0. To ensure numerical stability in Taubin filtering, the parameters must satisfy $0.0<\frac{1}{\lambda }-\frac{1}{v}<0.1$. In the present work, these parameters were set as λ=0.60, and v=0.635, with the number of iterations defined as n=20. Higher values of λ and n may compromise the fidelity of the original geometry, whereas ν influences the degree of bronchial shrinkage in the application of this filter (see Fig. 10).

Several quantitative metrics were applied to evaluate the final mesh quality, including minimum and maximum angles, edge lengths, and element-specific criteria such as the Jacobian and shape factor (Falsafioon et al., 2014). In this work, the minimum internal angle and shape factor were used as the primary quality metrics. The minimum angle criterion evaluated the quality of individual elements based on the smallest angle within the element. Elements exhibiting small angles are typically undesirable, as they may lead to numerical instabilities during both mesh generation and subsequent simulations. Larger internal angles are generally preferred to improve element quality. The shape factor, by contrast, quantifies how closely a given element approximates an ideal reference shape; in the case of the polygonal surface meshes used here, this reference is an equilateral triangle. Accordingly, the shape factor was calculated as defined in Eq. (14).

(14) $$SQ_{i}=\frac{4\sqrt{3}A_{i}}{\sum_{i=1}^{3}l_{i}^{2}},$$

where $A_{i}$ denoted the area of element i and $l_{i}(i=1,2,3)$ represented the lengths of its three edges. Elements with a shape factor closer to unity were considered more desirable, as they deviated less from the ideal equilateral shape.

### 2.5. Inlet and outlet processing

Following the mesh quality assurance of the ET and TB region airway segmentations, the next step in the workflow was to designate inlet and outlet surfaces, which should be planar surfaces to ensure numerical stability. To date, no automated framework for pre-processing outlets for CFPD simulations of the 3D human respiratory tract geometry has been published. In this work, an automated solution to address this challenge is presented. If done manually, the complexity and time needed for this task quickly escalate due to the exponential increase in the number of outlets corresponding to the bronchi generations, denoted as $2^{n\text{th}}+I$, where nth represents the number of bronchi generations, and I ranges from 1 to 3 based on the inclusion of the trachea, nasal cavity, or oral cavity. Consequently, as the bronchi generations increase, the number of outlets that need to be processed also rises significantly.

**Fig. 11.**
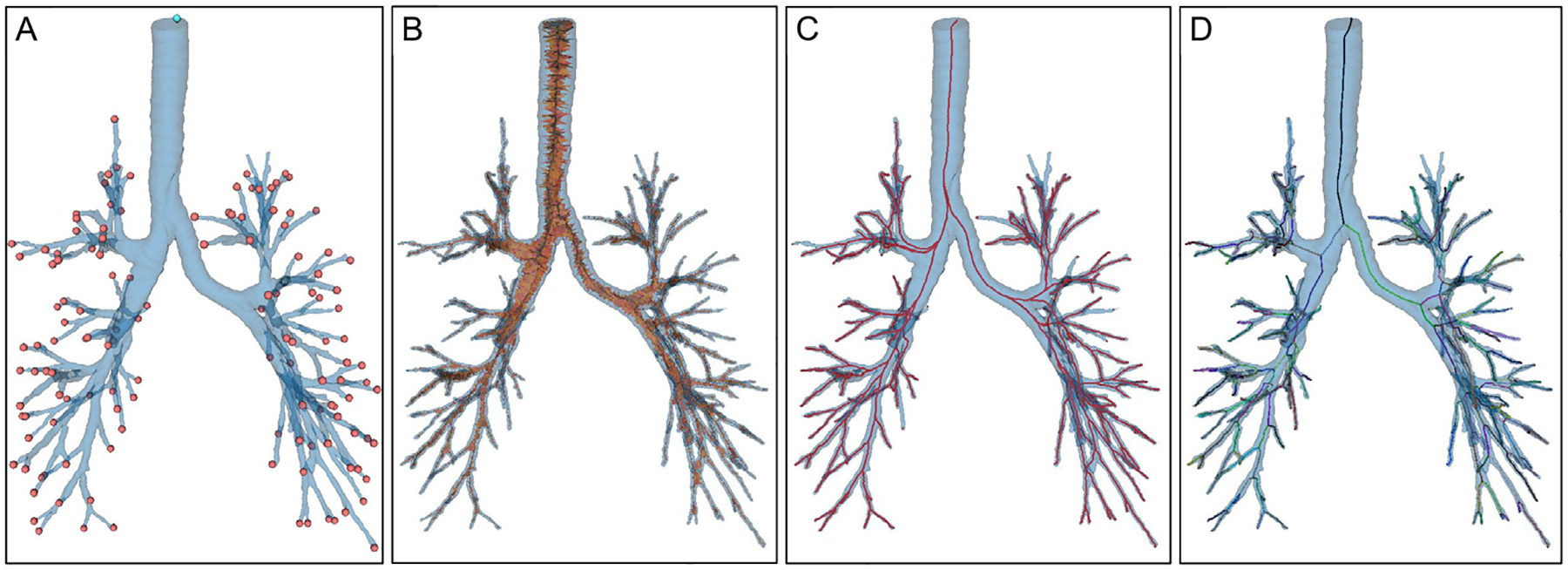
Steps for preprocessing the 3D airway geometry of the tracheobronchial region to produce a mesh appropriate for CFPD modeling. (A) Initial 3D surface mesh of the segmented airway, which contains multiple open ends corresponding to terminal bronchi. (B) Voronoi-based medial axis extraction to obtain a skeleton representation of the airway geometry, optimized to reside within the lumen of the airways. (C) Computed centerlines resulting from shortest-path calculations between endpoints using a weighted distance metric based on the maximal inscribed sphere radius, solved via the Fast Marching Method. (D) Final processed centerline network, where branches are organized into a hierarchy, smoothed, and resampled to define individual airway segments for further use in surface capping and mesh preparation.

Subsequently, a fully automated pipeline is presented to process the outlets and inlets of the reconstructed HRT, including the nostrils, oral cavity, trachea, or terminal bronchi, and prepare the model for CFD or CFPD simulations without manual intervention. This method comprised several steps, visually referenced in Fig. 11, starting with endpoint identification, centerline extraction, centerline segmentation, and surface capping.

The process involved extracting a medial axis skeleton from the input mesh, identifying airway branches, and capping each open end with a flat surface at the inlet and outlets. In the present work, the approach was to extract the centerlines of a 3D airway model using a Voronoi diagram guided by endpoints. Unlike curvature-based skeletonization, this method's computed centerlines reside within the vessel-like geometry's medial axis and were optimized for CFD applications as pointed out in Antiga et al. (2008).

A Voronoi diagram partitions space based on the closest proximity to a given set of seed points. The list of seed points is obtained using an automated method based on a network representation of the medial skeleton from the segmented HRT surface. Endpoints are nodes with only one neighbor in the extracted network. The inlet or starting point is determined based on the largest-radius criterion.

To compute the Voronoi diagram, using the same notation as established prior, let $𝒱\subset R^{3}$ be the set of vertices representing the airway surface mesh ℳ=(𝒱,ℱ). The Voronoi diagram 𝒱𝒟 of the domain $R^{3}$ concerning 𝒱 was defined as in Eq. (15).

(15) $$𝒱𝒟\left(p_{i}\right)=\left\{x\in R^{3}|d\left(x,p_{i}\right)\leq d\left(x,p_{j}\right),\forall p_{j}\neq p_{i},p_{j}\in 𝒱\right\},$$

where d(x,p) denotes the Euclidean distance.

Each point in the Voronoi diagram corresponds to the center of a maximal inscribed sphere. A maximal inscribed sphere $S_{i}$ centered at $x_{i}$ is a sphere that is not completely contained within any other sphere inscribed in the geometry, i.e.,

(16) $$S_{i}=\left\{y\in R^{3}|\left\|y-x_{i}\right\|\leq r_{i},\;\forall x_{j}\neq x_{i},r_{i}\geq r_{j}\right\}.$$

The radius $r_{i}$ contains the local vessel diameter and calculates the shortest path that adheres to the medial axis. The centerline is then computed as the shortest path between the endpoints of the airway. However, instead of computing geodesic distances directly, a weighted distance metric was introduced, where the path cost is inversely proportional to the maximal inscribed radius:

(17) $$C\left(p,q\right)=\int_{\gamma \left(p,q\right)}\frac{ds}{r\left(s\right)},$$

where γ(p,q) is the path between two endpoints p,q of the seed list, constrained to the Voronoi diagram, and r(s) is the radius of the maximal inscribed sphere along the path. To compute this weighted shortest path, the wave propagation was modeled using the Eikonal equation, $|\nabla T(x)|=\frac{1}{r(x)}$, where T(x) represents the arrival time of a wave propagating from a source point, and the speed of propagation is defined as the inverse of the maximal inscribed radius. This equation was solved numerically using the Fast Marching Method (FMM), which propagated a front from a source endpoint, recording the arrival time T(x) at every point of the Voronoi diagram.

Once the arrival times T(x) have been computed, the centerline was reconstructed by backtracking from the target point along the gradient of the arrival time:

(18) $$\frac{dx}{ds}=-\nabla T(x),$$

where s represents the parameter along the path, the extracted centerlines followed the steepest descent path along the weighted metric, maximizing the vessel diameter at every step. As a result, an example of the centerlines obtained are displayed in Fig. 11(C). The Vascular Modeling Toolkit (VMTK) implementation (Antiga et al., 2008) was used for the above-described steps.

The last step of the centerline extraction was to organize the airway centerlines into meaningful branching structure groups. From the output of the Voronoi-based centerlines, branch hierarchy is not explicitly defined; therefore, the VMTK vmtkCenterlineBranchExtractor was used to split the extracted paths into unique branches, each assigned a distinct identifier representing different airway segments. After all the branches were split, neighboring branches corresponding to the same airway branch were merged using vtkvmtkMergeCenterlines, which smooths transitions, removes unnecessary bifurcations, and resamples the paths at consistent intervals. The final airway centerline network structure, represented by different segments for each branch in each generation, can be seen in Fig. 11(D).

After obtaining the centerlines and endpoints, the final step in processing the reconstructed HRT geometry for CFD or CFPD simulations was to cap the inlets and outlets with planar surfaces. The general procedure used to cap the surface at each endpoint is outlined as follows:

1. Created a new STL object with a shape that resembles a thin cylinder. In practice, it was opted for a hexagon-shaped polygon for faster computation. The thickness of the new object should be greater than zero just to separate the two volumes, in the implementation 0.1 to 0.5 mm may be used.
2. Scaled the newly created object to cover the airway cross-section completely at the selected endpoint.
3. Moved the new object to match the endpoint position and aligned one face in the same direction as the centerline.
4. Capped the outlet using boolean difference operations. The body was split into two new bodies.
5. Kept the largest body and triangulated the newly created surface from the boolean operation. The surface at that endpoint is now flat and can be used as an outlet.
6. Repeat 1 to 5 for all the endpoints.

In more detail, the HRT capping process algorithm was implemented as follows. As previously defined, let the airway geometry be represented as a triangular surface mesh as ℳ=(𝒱,ℱ), with vertices $𝒱\subset R^{3}$, triangular faces ℱ, and edges ℰ. The endpoints of the centerline were identified as vertices with only one neighbor, but this time, they were from the newly refined centerline instead of the medial axis skeleton. Formally, these endpoints were defined as ${ℰ}_{C}=\{p\in 𝒞|\text{deg}(p)=1\}$, where 𝒞 was the set of centerline points and deg(p) denoted the degree (number of adjacent vertices) of the point p.

For each endpoint $p_{b}\in {ℰ}_{C}$, a polyhedron geometry was introduced to cut the geometry at that endpoint and cap the opening to be used as an outlet. The first step was to construct a polyhedron that approximated a thin cylinder, since it matched the cross-section shape of the HRT at that location, typically the bronchi, trachea, oral cavity or nostrils. In the actual implementation, an hexagon-like polyhedron with a thin thickness t was chosen for computational efficiency (t was 0.1 to 0.5 mm thick in the implementation). For the description and definition of the presented algorithm, a thin cylinder with thickness t polyhedron aligned with the z-axis was described as in Eq. (19) to represent the polyhedron.

(19) $$C_{\text{cap}}=\left\{x\in R^{3}\left|\sqrt{{\left(x-p_{b}\right)}_{x}^{2}+{\left(x-p_{b}\right)}_{y}^{2}}\leq r_{b},\;\right|{\left(x-p_{b}\right)}_{z}\left|\leq \frac{t}{2}\right.\right\}$$

where $p_{b}$ is one endpoint position on the centerline, $r_{b}$ is the radius that completely covered the local airway cross-section, taken as the maximal inscribed sphere radius at $p_{b}$, and $n_{b}$ is the local normal vector approximating the airway cross-section orientation, obtained from the tangent direction of the centerline at $p_{b}$:

(20) $$n_{b}=\frac{p_{b}-p_{\text{next}}}{\left\|p_{b}-p_{\text{next}}\right\|},$$

with $p_{\text{next}}$ being the nearest neighboring point along the centerline.

The body $C_{\text{cap}}$ was then aligned to match the cross-section of the airway by applying a rigid-body rotation. The rotation aligned the cap's normal vector to match the centerline's tangent vector $n_{b}$. The rigid body transformation was based on the Rodrigues rotation formula, using the implementation from scipy.spatial.transform.Rotation from Scipy (Virtanen et al., 2020), described in Eq. (21).

(21) $$R=I+\text{sin}(\theta )[a{]}_{\times }+(1-\text{cos}(\theta ))[a{]}_{\times }^{2},$$

where $\theta =\text{arccos}\left(n_{b}\cdot z\right)$, with z being the initial cap normal, and $[a{]}_{\times }$ is the skew-symmetric cross-product matrix corresponding to the rotation axis $a=z\times n_{b}$.

**Fig. 12.**
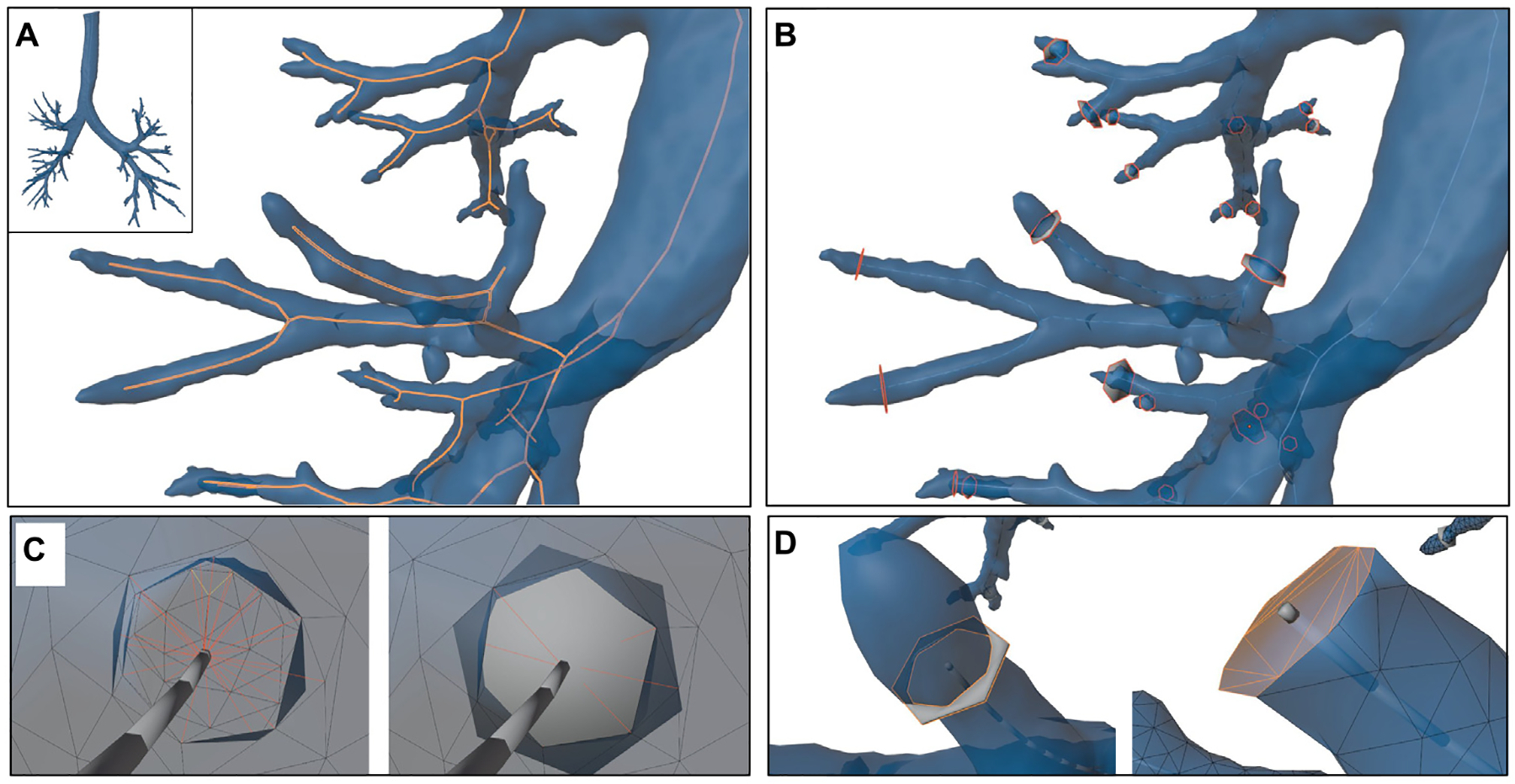
Detailed steps for outlet capping procedure for preparing airway surface meshes for CFD or CFPD simulation. (A) Initial 3D airway surface mesh with multiple open endpoints identified from centerline extraction. (B) Creation of a thin hexagonal body aligned with the local centerline direction at each endpoint. (C) Detail on the cap scaling to fully intersect the airway cross-section at the endpoint. From the selected point, the associated vertices are used to scale the surface for the cap. (D) Boolean subtraction between the original airway mesh and the cap geometry, resulting in a planar cut. The largest enclosed volume is retained, and the resulting open surface is triangulated to form a flat, CFD-compatible outlet boundary. This procedure is repeated at each identified endpoint to generate a watertight mesh with planar inlet and outlet surfaces.

After orientating the cap and positioning it at the endpoint, the next step was to perform a boolean subtraction operation on the original mesh ℳ using the aligned cap $C_{\text{cap}}$. The Boolean operation divided the original HRT mesh into two separate bodies. The largest resulting body, in terms of enclosed volume, was retained, and the newly created planar boundary surface from the Boolean subtraction was triangulated to comply with the STL format, as depicted in Fig. 12(D). Following this operation, the capped airway endpoint became planar, geometrically well-defined, and compliant with CFD or CFPD boundary condition assignment.

The capping procedure defined prior was systematically repeated for all endpoints identified in the refined centerline. After completing the procedure, the most significant capped boundary was set as the inlet by computing and comparing their surface areas. The final result was a watertight triangular mesh geometry, prepared with explicitly defined inlet and outlet surfaces, and compatible with CFD and CFPD simulations.

Automating the entire pipeline eliminated the need for manual identification and capping of outlets, significantly reducing processing time and minimizing human error. Centerline extraction not only facilitated the localization of inlets and outlets but also ensured that the capping surfaces were properly aligned with the airway's anatomical structure. This fully automated framework enabled reproducible and pre-processing of anatomically complex airway geometries for use in CFD simulations.

### 2.6. CFPD modeling

#### Methods

Once the surface mesh of the HRT had been processed to be CFD compliant, the subsequent step involved configuring the volumetric mesh, airflow model, particle-flow interactions, boundary conditions, and solver-specific input files for the CFD or CFPD simulation.

This Subsection outlines a fully automated workflow for meshing and executing the CFPD simulations. While multiple CFD solvers have been developed throughout the years with comparable capabilities for this application, this study employed OpenFOAM, initially inspired by Weller et al. (1998) as an open-source solution, while StarCCM+ (Siemens Digital Industries Software, 2021) was used as a licensed choice of the solver. A Python framework was developed to automate the process from end to end for OpenFOAM simulations. On the other hand, for StarCCM+ simulations, a Java script was developed using StarCCM+ APIs. The StarCCM+ automation was separated into two parts — one for computing the airflow alone and the other for particle coupling. In OpenFOAM, the SnappyHexMesh tool was used for body-fitted mesh generation, while in StarCCM+, the polyhedral meshing tool was used. For the airflow field, a Reynolds-Averaged Navier–Stokes (RANS) model was applied; specifically, the k-ω SST LM model was validated for particle deposition in both upper and lower HRT simulations (Koullapis et al., 2018). The boundary conditions for this model were carefully considered to ensure model convergence and numerical stability, including parameters for pressure, turbulent kinetic energy (k), and turbulent specific dissipation rate (ω). Particle-flow coupling in OpenFOAM employed a pseudo four-way coupled Lagrangian approach using the Multi-Phase Particle In Cell (MPPIC) solver. In contrast, in StarCCM+, a two-way coupled Lagrangian tracing method with the Lagrangian Multi-phase (LMP) solver was used. OpenFOAM automation scripts were used to initialize all necessary parameters and dictionaries for the MPPIC solver. Similarly, StarCCM+ used Java code to automate the simulation setup, including importing geometry, assigning patches, and applying turbulence models, particle interactions, boundary, and initial conditions.

In detail, for the meshing process, a hexahedral-based mesh for OpenFOAM and a polyhedral mesh for StarCCM+ were employed. The difference between a hexahedron-dominant mesh and a polyhedral mesh did not affect the PDPs, as discussed by Thomas and Longest (2022), demonstrating no appreciable differences in deposition efficiency values using a simplified bifurcation geometry. To generate the volumetric mesh in OpenFOAM, the SnappyHexMesh utility was used to generate a body-fitted mesh. An array of parameters was tested to propagate the boundary layer well through the geometry, achieve this efficiently, and maintain mesh quality during the process. Findings indicated that the most relevant part is starting with a well-posed background mesh, where all the elements conform to a cuboid geometry; this avoided a high skewness later and helped propagate the boundary layer. To achieve such background mesh, the 3D HRT is bounded in a box, and then the box is divided an integer number of times to fit cubic elements. The size of the cubic element is taken according to the size of the minor outlet, therefore having at least one element intersecting each surface, as recommended by the SnappyHexMesh user manual (OpenFOAM, 2025). If the size of the minor outlet exceeds 1.5 mm, we set the cubic element to be 1.5 mm, based on mesh independence studies performed. In StarCCM+, the element base size is a global parameter that sets the initial tetrahedral cell size before any refinement to a polyhedral mesh and application of local sizing controls. A table with key parameters to render a suitable mesh using SnappyHexMesh in OpenFOAM is provided in Table 2. Note that only non-default parameters are listed; the rest of the parameters used were the default parameters.

A RANS approach was used to model the turbulence. In particular, the k-ω SST LM model (Langtry & Menter, 2009) was selected to capture transitions from laminar to turbulent flow in human airways. This model demonstrated agreement with higher-order CFPD simulations and in-vitro experiments for particle deposition by Koullapis et al. (2018) in the ET and TB regions of the HRT. In their study, Koullapis et al. (2018) used Large Eddy Simulation turbulence models to compare against different RANS models and validated the CFPD-predicted particle deposition using previous in vitro experiments performed on the same airway geometry. This model was also employed by Zhang and Kleinstreuer (2011) and Shang et al. (2019) in independent studies on the HRT. The boundary conditions for pressure, turbulent kinetic energy (k), and turbulent specific dissipation rate (ω) were carefully defined, as these conditions strongly affected the model convergence.

**Fig. 13.**
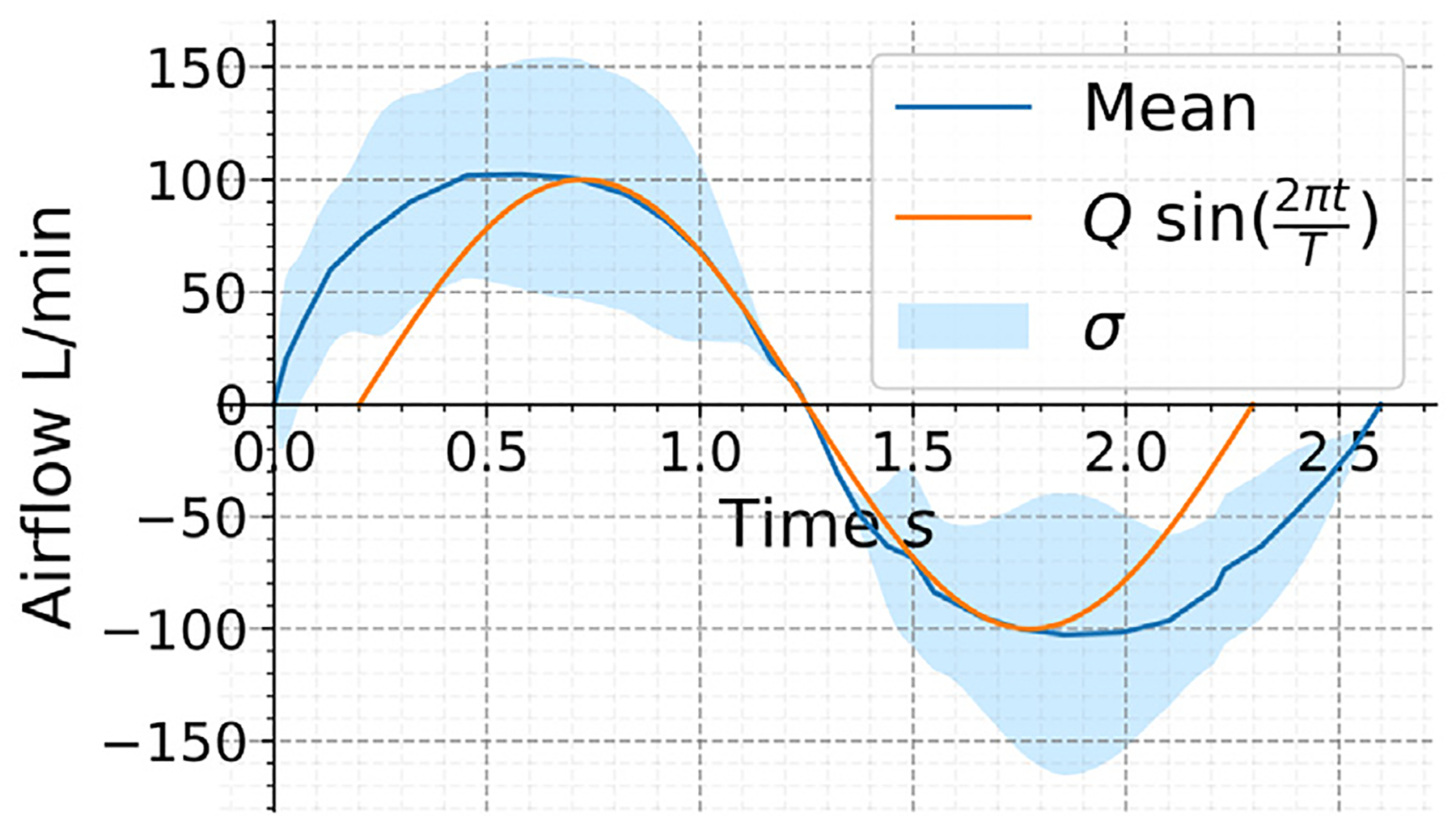
Airflow for a respiratory cycle for the mean population and its standard deviation σ using Silverman et al. experimental data (Silverman et al., 1951). A negative airflow in this context represents exhalation, and a positive airflow means inhalation. An approximation to the mean respiratory cycle by a sin function is plotted in orange. The blue line represents the mean values from the original experimental data, and the shade represents the standard deviation.

The boundary conditions for the velocity u were selected to represent a respiratory cycle of a human subject under heavy-exercise breathing conditions while balancing the computational costs. The average respiratory cycle with its standard deviation was represented in Fig. 13, based on experimental results from Silverman et al. (1951). A sin function was used to approximate the average respiratory cycle, and to reduce computational overhead, a shorter period of T=2 seconds was adopted.

For a given flowrate $Q(t)=Q_{\text{max}}\text{sin}\left(\frac{2\pi t}{T}\right)$, the inlet area in each geometry was computed, and the magnitude of the inlet velocity was calculated using $\|u\|=\frac{Q}{A}$. In OpenFOAM, this was implemented through custom boundary conditions, while StarCCM+ field functions served the same purpose. While the flow rate can be changed to represent any breathing condition, users must ensure that the flow rate used is appropriate for the turbulence model and assumptions made in the boundary conditions. For example, at low flowrates (i.e., resting breathing condition), turbulence modeling is not approximate, and a laminar airflow model should be applied.

Particle-flow coupling employed a four-way coupled Lagrangian approach in OpenFOAM using the Multi-Phase Particle In Cell (MPPIC) solver. On the other hand, in StarCCM+, a two-way coupled Lagrangian tracing was implemented using a Lagrangian Multi-phase (LMP) solver. MPPIC and LMP solvers account for drag, lift, virtual mass, and gravity forces. However, in the StarCCM+ LMP solver, an additional force was included using the user-defined volumetric forces to account for the Brownian diffusion for submicron particle transport.

Eq. (22) defines the transport of particles, where $C_{d}$ is the drag coefficient of the particle, dependent on the chosen drag model. In this work, the Stokes-Cunningham correlation was applied for solid particles was implemented using field functions in StarCCM+, while in OpenFOAM the Stokes-Cunningham is natively implemented. The default drag model for solid particles in StarCCM+ (Schiller-Naumann) does not incorporate non-continuum slip corrections relevant for submicron particles. This limitation was addressed by applying the Cunningham slip correction factor. The airflow velocity is denoted by u and the particle volume fraction by $\alpha_{p}$, which was negligible for the conditions simulated. Particle's mass is denoted by $m_{p}$.

(22) $$m_{p}\frac{\partial v_{p}}{\partial t}=\frac{1}{2}C_{d}\rho A_{p}\left|v_{s}\right|v_{s}+m_{p}g\left(1-\alpha_{p}\right)+f_{\text{Brownian}}+f_{\text{lift}}+f_{\text{virtual}\;\text{mass}}$$

where $C_{d}$ is the drag coefficient of the particle, ρ is the density of the continuous phase, $v_{s}=u-v_{p}$ is the particle slip velocity, with u being the instantaneous velocity of the continuous phase. The $A_{p}$ is the projected area of the particle, g is the gravitational acceleration, $f_{\text{Brownian}}$ is the force due to Brownian motion, $f_{\text{lift}}$ is the lift force, and $f_{\text{virtual}\;\text{mass}}$ is the virtual mass force.

The Brownian force $F_{B}$ expression for a particle in a fluid medium as defined by Li and Ahmadi (1992) is expressed in Eq. (23) and can be used for both laminar and turbulent flow regimes.

(23) $$F_{B}=\sqrt{\frac{2k_{b}T_{o}\alpha }{\Delta t}}\xi$$

where $k_{b}$ is the Boltzmann constant, $T_{o}$ is the temperature of the fluid in Kelvin, α is the particle's mobility, Δt is the time step, and ξ is a vector of Gaussian random numbers with zero mean and unit variance that will define the direction of the force. The particle mobility α is a measure of how easily a particle moves when subjected to a force and is defined as $\alpha =\frac{D}{kT}$ where D is the diffusion coefficient. The diffusion coefficient D can be calculated using the Einstein relation for spherical particles: $D=\frac{kT}{3\pi \eta d}$, where η is the air viscosity and d is the particle diameter.

Boundary conditions for pressure were assigned as fixed-value conditions at outlets and zero-gradient (Neumann) conditions at walls and inlets. For the inlet turbulent kinetic energy (k), an assumption of isotropic turbulence was used, with $k=\frac{3}{2}{\left(I\left\|u_{\text{ref}}\right\|\right)}^{2}$, where the turbulence intensity I was set to 4%, corresponding to medium turbulence conditions. The reference velocity $u_{\text{ref}}$ was set to the peak inhalation velocity, which varied depending on the geometry, as the flow rate was specified as a fixed quantity. For the inlet turbulent specific dissipation rate (ω), the expression $\omega =\frac{k^{0.5}}{C_{\mu }^{0.25}L}$ was applied, with the constant $C_{\mu }=0.09$, and a characteristic length scale L defined by the hydraulic diameter $d_{h}$ of the inlet. At outlets, zero-gradient boundary conditions were imposed for both k and ω. In StarCCM+ software, the wall treatments were handled using the “All y+” feature; in OpenFOAM, manual handling for the wall functions was required. Thus, the kLowReWallFunction was used for turbulent kinetic energy and serves both low- and high-Reynolds regimes. For ω, the standard omegaWallFunction was employed; this combination resulted in good numerical stability within the SST transitional turbulence modeling.

As a point of clarification, despite the name of kLowReWallFunction, it provides appropriate boundary constraints on the turbulent kinetic energy in low and high Reynolds number flow regimes.

The RANS k-ω SST Langtry-Menter turbulence transition model had additional equations for intermittency (γ) and transition momentum thickness Reynolds number (${Re}_{\theta }$). Inlet boundary conditions were set as: γ=1, and ${Re}_{\theta }$ calculated according to Eq. (24), where turbulence intensity (Tu) was defined as $Tu=100\frac{\sqrt{2/3k}}{\left\|u_{\text{ref}}\right\|}$. Neumann boundary conditions ($\frac{\partial \gamma }{\partial n}=0,\frac{\partial {Re}_{\theta }}{\partial n}=0$) were applied at walls and outlets.

(24) $${Re}_{\theta }=\left\{\begin{array}{ll} 1173.51-589.428Tu+\frac{0.2196}{{Tu}^{2}}, & \text{if}\;Tu\leq 1.3\\ \frac{331.5}{(Tu-0.5658{)}^{0.671}}, & \text{if}\;Tu>1.3 \end{array}\right.$$

With well-established turbulence models, the CFPD simulation setup was automatized for StarCCM+ and OpenFOAM. In OpenFOAM, a Python script initializes all necessary dictionaries, including the velocity (u), turbulence kinetic energy (k), specific rate of dissipation (ω), turbulent viscosity ($v_{t}$), pressure (p), and intermittency ($\gamma_{int}$), as well as Reynolds shear stress ($Re_{\theta t}$). The script also generates dictionaries required for the MPPIC solver's colliding cloud model.

A summary of the models, parameters, and values used in this work is tabulated in Table 3, modified from Bartol, Graffigna Palomba et al. (2024).

The treatment of particles that exited the airway geometry during inhalation depended on the particle size distribution used in the simulation. For monodisperse particle distributions, particles escaping through the outlets were assumed to have the same properties, and exhalation could be modeled by re-injecting particles uniformly across all outlets using a single particle size. However, for polydisperse distributions (e.g., log-normal), re-injection during exhalation was not performed. This decision was based on the complexity of tracking and reconstructing outlet-specific particle size distributions across a large number of outlet surfaces—typically 128 to 256 in our geometries. Implementing a separate surface monitor and injector for each outlet would be required, and the resulting particle sampling demands would make the simulation computationally intractable. Therefore, in polydisperse cases, particles that exited through the outlets during inhalation were not reintroduced into the domain for the exhalation phase.

The CFPD models presented in this study do not resolve deposition in the pulmonary region of the human lung due to limitations in airway geometry segmentation. Therefore to address this limitation, this research used a hybrid strategy that leverages established ICRP Publication 66 and ICRP Publication 130 models to estimate deposition in the distal pulmonary region, from in-house developed tools from the group in Mate-Kole et al. (2023). While extending the segmented geometries with the idealized airway model described by Yeh and Schum (1980a), up to G23 was considered, this approach proved computationally intensive and challenging to automate across different cases while maintaining anatomical continuity. Instead, this research opted to implement a correction step using deposition factors from ICRP Publication 66 and 130 to approximate particle behavior in the pulmonary region of the lung.

In particular, particles that escaped deposition in the resolved geometry of the CFPD simulation were tallied and adjusted using the ICRP-derived correction factor, denoted as ${\text{ICRP}}_{\text{correction}}$. This term quantifies the expected fraction of particles that would deposit in the alveolar-interstitial (AI) region and small conducting airways (i.e., bronchioles beyond the resolution of the CT-based segmentation, typically part of the bb region, as defined in ICRP Publication 66) based on particle properties (aerodynamic diameter, density, particle size and distribution), breathing parameters (tidal volume, breathing frequency), and airway region-specific deposition efficiencies provided in ICRP Publication 66 and ICRP Publication 130. To compute the overall particle deposition, Eq. (25), the correction is applied by multiplying the escaped particle count by a region-specific deposition efficiency, accounting for the portion of the non-deposited particles into the unresolved regions.

(25) $$\text{nDF}=\frac{n_{\text{Stick}}+n_{\text{Scape}}\times {\text{ICRP}}_{\text{correction}}}{n_{\text{Total}}}$$

In Eq. (25), $n_{\text{Stick}}$ represents the number of particles that stuck (i.e., deposit without removal or re-suspension) to the walls of the respiratory tract, $n_{\text{Scape}}$ represents the number of particles that escape deposition, ${\text{ICRP}}_{\text{correction}}$ is a correction factor from the ICRP accounting for the alveolar region not modeled in the defined geometry, and $n_{\text{Total}}$ is the total number of particles in the CFPD simulation.

This correction method was chosen for its computational efficiency in estimating the complete deposition profile across the pulmonary region of the HRT. The assumption is that, a priori, the CFPD results in the idealized airway model described by Yeh and Schum (1980a) should relate to the ones computed from ICRP. While this approach does not simulate particle trajectories in the alveolar spaces directly, the ICRP model has been extensively validated and remains a standard in both dosimetric and risk assessment frameworks.

#### Results

Extensive verification and validation were conducted in different meshes and geometries, as detailed in the Supplemental Material from Bartol, Graffigna Palomba et al. (2024). The verification and validation process demonstrated that the CFD/CFPD automated workflow generated mesh-independent results in alignment with the NPARC Alliance guidelines (Slater et al., 2000). Seven progressively refined meshes were analyzed under steady-state conditions at an airflow rate of $Q=90\;L{\;\text{min}}^{-1}$. Mesh quality was carefully controlled, achieving skewness values consistently below 0.3, reflecting the high-quality meshing techniques. The computed order of grid convergence ($p_{c}$) for pressure and turbulent kinetic energy was 1.61 ± 0.30 and 1.66 ± 0.17, respectively, which aligns with expectations for second-order discretization schemes.

The Grid Convergence Index (GCI) evaluation, based on three representative meshes with refinement ratios close to 2, also demonstrated that discretization errors were well within acceptable limits. The fine-grid GCI was less than 0.46% for pressure and 3.22% for turbulent kinetic energy, confirming that the solutions were within the asymptotic convergence range.

**Fig. 14.**
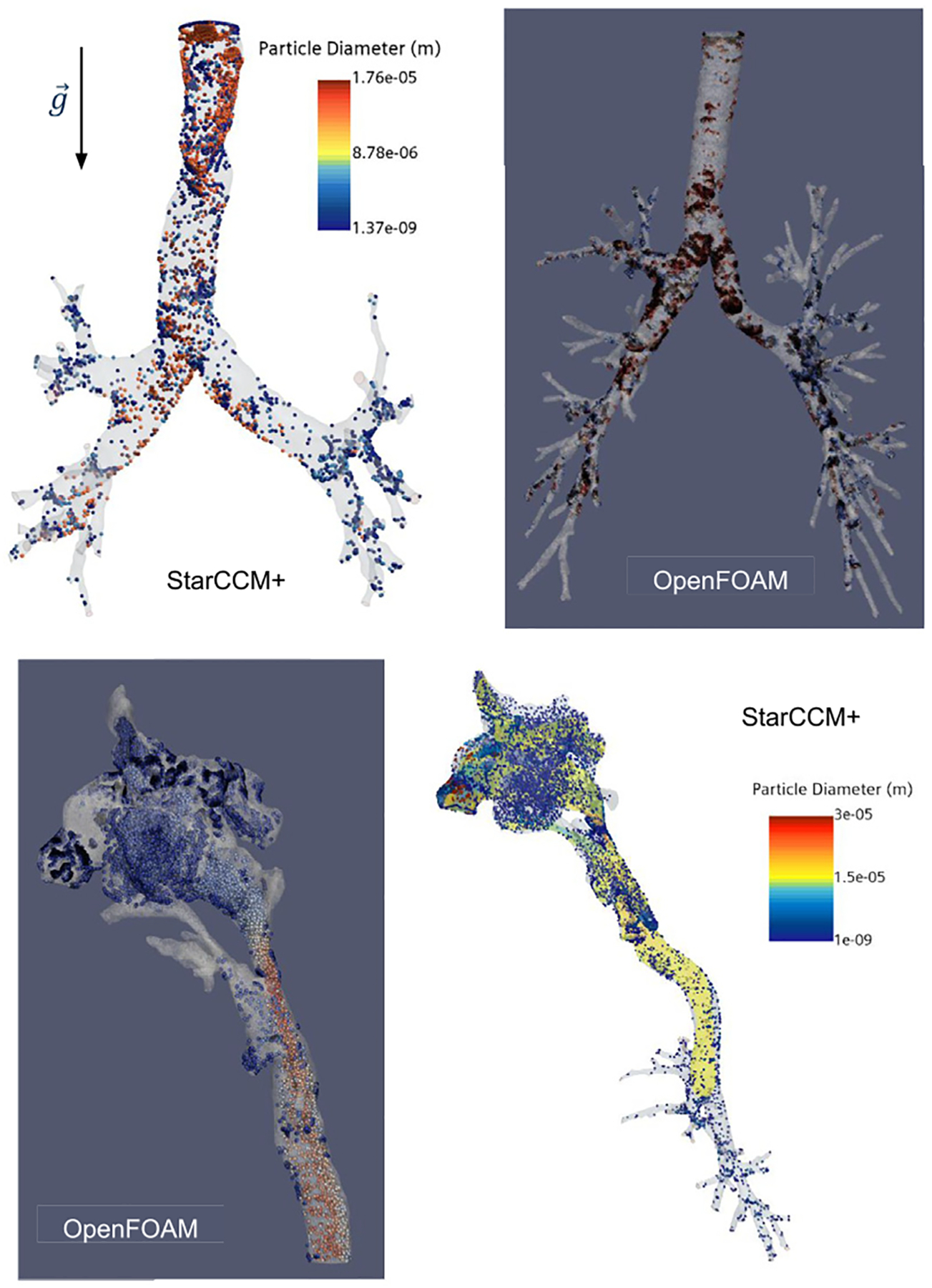
Exemplary results from the automated pipeline for StarCCM+ and OpenFOAM solvers. The figure shows PDPs for two tracheobronchial regions of the HRT, one extrathoracic region of the HRT, and one full HRT, combining both regions.

The output of the CFPD simulation was the PDPs across the HRT, which were computed for the upper, lower, or full reconstructed HRT depending on the geometry employed. An example of the PDP output is shown in Fig. 14, where both PDPs results from OpenFOAM (using ParaView as visualizer) and StarCCM+ are shown at the end of one respiratory cycle. A comprehensive analysis of PDPs across multiple geometries, validation against prior studies, and comparison of one-way versus two-way coupling is provided in Bartol, Graffigna Palomba et al. (2024). In the study conducted by Bartol, Graffigna Palomba et al. (2024), a heavy-breathing respiratory condition simulated, where airborne iodine particles were selected as the source, modeled as a log-normal particle size distribution with a mean size of 0.42μm and a geometric standard deviation of σ=3.5.

#### 2.6.1. Limitations

The implemented RANS k-ω SST Langtry-Menter turbulence model, while validated for human respiratory tract airflow, inherently relies on empirical relationships and assumptions for turbulence transition through the γ parameter, which governs the laminar, transitional, or turbulent state of flow. This turbulence model was validated against LES data for upper airway and proximal tracheobronchial regions (Koullapis et al., 2018), but has not yet been validated for highly distal bronchi or for low airflow rates, where a laminar modeling may be more appropriate.

Moreover, the PDPs predicted by CFPD is sensitive to boundary layer mesh quality. While rigorous mesh convergence and quality assessments were conducted, the automated meshing strategy, whether using SnappyHexMesh (OpenFOAM) or polyhedral meshing (StarCCM+), may still encounter difficulties in maintaining boundary-layer integrity in complex airway geometries, especially around bifurcations and sharp curvature regions. Small geometric imperfections, such as sharp non-physical edges not captured in the post-processing, can introduce locally skewed cells, potentially affecting local deposition behavior and introducing numerical instability.

Another limitation concerns the airflow representation used in simulations. The workflow implemented a simplified sinusoidal breathing pattern representative of heavy exercise conditions, providing a general pattern for most of the population. However, subject-specific human breathing patterns vary in frequency, amplitude, and asymmetries, which must be considered in subject-specific analyses. Therefore, caution is advised when interpreting results from highly idealized breathing cycles in applications such as inhalation therapies.

### 2.7. Monte-Carlo model definition

#### 2.7.1. Methods

**Fig. 15.**
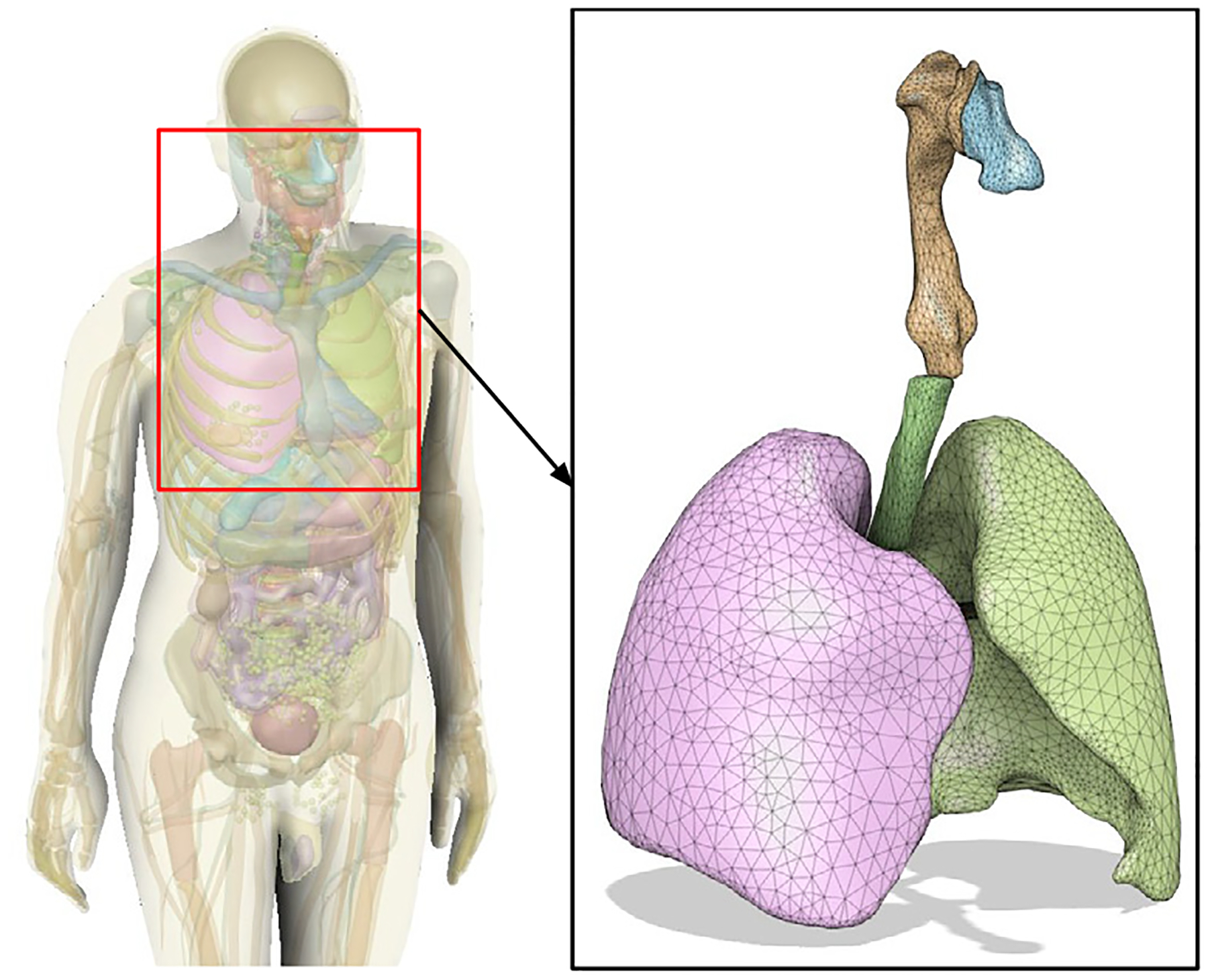
Torso of the adult male MRCP phantom (left) and a detail of the HRT (right) modeled by Kim et al. (2020) in the MRCP.

**Fig. 16.**
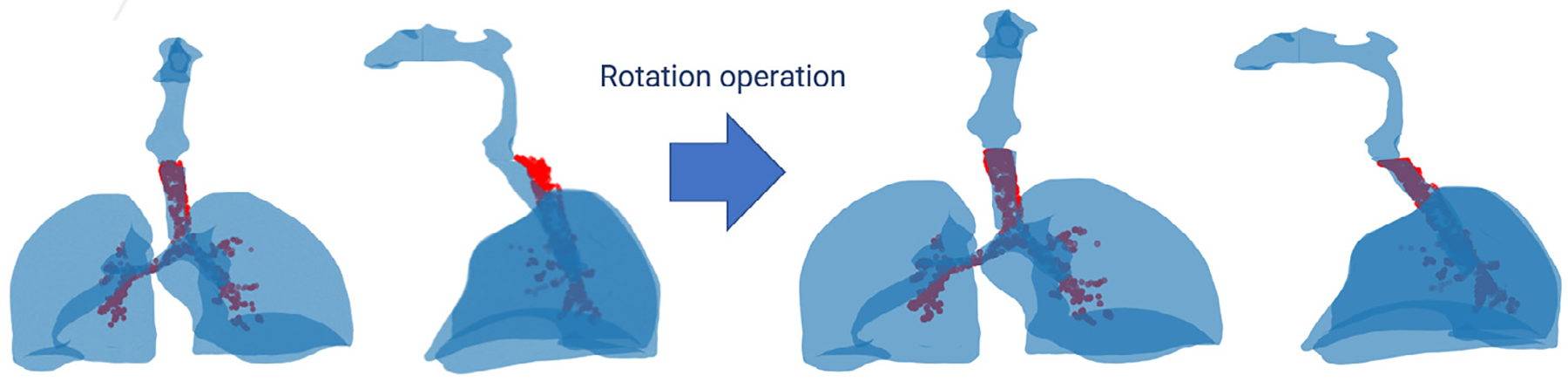
Superposition of the particle deposition profiles (PDPs) obtained via Computational Fluid and Particle Dynamics (CFPD) simulations, on the Human Respiratory Tract (HRT) modeled in the Mesh-type Reference Computational Phantom (MRCP) used in this work. Original (left) and post-processed (right) particle distribution profiles from the CFPD simulations overlapped in the HRT and lungs male MRCP phantom geometry.

Using the detailed PDPs obtained from the CFPD simulations, a coupling to calculate the absorbed dose from radioactive source particles were incorporated into the workflow. In case the inhaled particles are radioactive, the user can be interested in computing the dose imparted from the particles to different organs. For this purpose, Particle and Heavy Ion Transport code System (PHITS) from Sato et al. (2013) Monte Carlo software was used in conjunction with the Adult mesh-type reference computational phantom (MRCP) developed by Kim et al. (2020). The phantom and a detail of the HRT are shown in Fig. 15. A Python tool was developed to align the particle distribution from the CFPD simulations with the HRT in the MRCP phantom. In Fig. 16, the original particle distribution is overlaid with the HRT of the MRCP phantom is displayed. The tracheal carina was used as the point of reference to align the CFPD particle distribution with the MRCP phantom to match the spatial location. Subsequently, rigid-body rotation operations were applied to the distribution to ensure proper alignment with the MRCP phantom's tracheal axis.

Each particle was treated as a point source, with its source activity proportional to the particle mass. Since PHITS is limited to handling no more than 500 point sources per simulation, the simulation was divided into multiple batches, each containing a maximum of 500 point sources. In the cases involving double decay modes (i.e., coupled beta and gamma decay), each particle was represented by two point sources — one for the γ decay and one for the $\beta^{-}$ decay. As a result, only 250 particles could be simulated per batch under these conditions. The activity for each particle was assigned to be proportional to its volume, meaning $A_{i}=\frac{V}{a}\rho$, where a is the specific activity in [Bq kg^−1^], ρ is the particle density [kg m^−3^], and V is the particle's volume [m^3^].

#### 2.7.2. Results

In this study, building on the results from Bartol, Graffigna Palomba et al. (2024), the I131 was selected for internal dosimetry simulations due to its dual radioactive decay beta and gamma source emissions and a half-life of approximately 8.02 days. The particle distribution profiles in the TB airways were derived from the CFPD simulation described in Section 2.6, where airborne particles of I131 were simulated as a log-normal distribution with a mean size of 0.42μm and a geometric standard deviation of σ=3.5). The respiratory conditions corresponded to heavy-breathing, with a flowrate given by $Q(t)=Q_{\text{max}}\text{sin}\left(\frac{2\pi t}{T}\right)$, where $Q_{\text{max}}=100\;L/\text{min}$, yielding an average flowrate of ⟨Q(t)⟩≃63L/min. In practice, any radionuclide included in PHITS libraries can be selected for implementation in the prescribed workflow. In this study, 1154 deposited particles were modeled as point sources, resulting in five simulation batches. The absorbed dose to various organs was estimated and compared to a reference case involving a uniform particle distribution source, where the same total activity was uniformly distributed across the trachea and BB region. The particle mass specific activity was set to $a_{p}=1\times 10^{12}\frac{Bq}{Kg}$, resulting in a total activity of 17.48Bq, partitioned as 8.742 Bq in the BB region and 8.735 Bq in the trachea. Table 4 summarizes the differences in the absorbed dose across selected organs when using the CFPD-informed particle distribution compared to a uniform particle distribution. The largest discrepancies in dose were observed in the lungs, trachea and BB region, which are either in proximity to source-bearing organs contained the radioactive sources in situ.

#### 2.7.3. Limitations

Several inherent limitations must be addressed before subject-specific radioactive aerosol dosimetry can be fully achieved. Primarily, the methodology relies heavily on alignment and positioning of the particle distribution derived from CFPD simulations with the HRT of a population-percentile-specific MRCP phantom. Discrepancies between the geometry used for the CFPD simulations and the airway geometry in the MRCP phantoms may lead to point sources being placed outside the phantom airway structure. Moreover, a rigid transformation does not accommodate more subtle anatomical changes, which derive in these local deviations in the spatial distribution of particles and consequently, the absorbed dose estimation. While a deformable image registration could be done, the number of bronchi generations included in the MRCP phantom extends only to the 2nd generation (G2); therefore, this solution is not as feasible.

Moreover, assuming each particle as a discrete point source with activity proportional to its mass or volume may oversimplify the physical characteristics of radioactive particles, particularly for larger particles or clustered aerosol particles. In realistic scenarios, particle shape, and spatial clustering can significantly influence local energy deposition due to the self-shielding effects.

Lastly, real inhalation events rarely involve a single respiratory state, typically encompassing varying breathing dynamics. Thus, a particle distribution from a CFPD simulation using a single state for the respiratory condition represents only an approximate baseline that may not reflect physiologically realistic exposure conditions, thereby limiting applicability to complex exposure scenarios.

### 2.8. CT datasets

The anonymized CT images for this study were obtained from publicly available sources. For the lower respiratory tract, the same databases used in Bartol, Graffigna Palomba et al. (2024) were employed in this study:

- From the EXACT'09 challenge (Lo et al., 2012) 46 de-identified chest CT scans were obtained (From the trachea up to the 5th to 7th generation of bronchi depending on the quality of the CT scan). The conditions of the scanned subjects varied widely, from healthy volunteers to subjects with severe abnormalities in the airways or lung parenchyma.
- Pediatric Chest/Abdomen/Pelvic CT Exams with Expert Organ Contours (Pediatric-CT-SEG) (Jordan et al., 2022, 2021) from The Cancer Imaging Archive (TCIA) (Clark et al., 2013) represent 359 random pediatric cases based upon routine clinical indications. No medical or diagnostic data are available for any subject dataset.
- A Large-Scale CT and PET/CT Dataset for Lung Cancer Diagnosis (Lung-PET-CT-Dx) (Li et al., 2020) from TCIA (Clark et al., 2013). All the 355 subjects in the database were diagnosed with Adenocarcinoma, Small Cell Carcinoma, Large Cell Carcinoma, and Squamous Cell Carcinoma.
- Chest Imaging with Clinical and Genomic Correlates Representing a Rural COVID-19 Positive Population (COVID-19-AR) (Desai, Baghal, Wongsurawat, Al-Shukri et al., 2020; Desai, Baghal, Wongsurawat, Jenjaroenpun et al., 2020; Jenjaroenpun et al., 2021) from TCIA (Clark et al., 2013) includes data collected from 105 hospitalized subjects with a positive COVID-19 laboratory test verified diagnosis, with imaging studies conducted within eight days prior to diagnosis and at least one imaging study post-diagnosis.
- CT Ventilation as a Functional Imaging Modality for Lung Cancer Radiotherapy (CT vs. PET Ventilation Imaging) (Eslick et al., 2018, 2022) from TCIA (Clark et al., 2013) includes health information of 16 subjects all of whom had mild, moderate, or severe chronic obstructive pulmonary disease and impairment in the diffusing capacity of the lungs for carbon monoxide.

To investigate the upper respiratory tract — which encompasses the trachea, pharynx, oral cavity, and nasal cavity — 111 de-identified head-neck CT scans from the TCIA archive (Clark et al., 2013) were acquired, specifically sourced from the Head-Neck Cetuximab database (Ang et al., 2014; Bosch et al., 2015).

## 3. Conclusions

This study developed an end-to-end automated framework for generating personalized PDPs in the HRT and computing absorbed dose in scenarios involving inhaled radioactive particles. The presented pipeline addresses limitations associated with traditional geometric modeling and establishes a reproducible methodology suitable for conducting subject-specific internal dosimetry. Using CT imaging data, volumetric segmentations were produced, from which surface meshes were extracted and refined. The resulting 3D geometries were subsequently processed to meet the quality and consistency requirements of CFD and CFPD simulations. This process included multiple mesh repair, quality control, and automated identification of inlets and outlets.

Preparing a geometry for a CFD/CFPD simulation requires adherence to the most stringent quality criteria within the modeling field. Accordingly, multiple steps were implemented to verify mesh fidelity. High-fidelity CFPD simulations were then conducted using automated mesh techniques and pre-converged solvers adapted to individual HRT anatomical configurations. The final step in the pipeline involved automated Monte Carlo simulations to compute spatially resolved energy deposition within mesh-based phantoms for inhalation scenarios.

This study provided a novel and comprehensive approach encompassing geometry reconstruction, preprocessing, CFPD modeling, and Monte Carlo simulations. The CT-derived HRT geometries, efficiently captured complex anatomical structures through a combination of deep learning and traditional refinement algorithms. For CFD/CFPD workflows, both an open-source option (OpenFOAM) and a commercial solver (StarCCM+, Siemens) were implemented, allowing flexibility in software infrastructure.

A key strength of the framework is its modular structure and computational efficiency. The modularity allows users to initiate the workflow from multiple entry points — including a CT scan, a raw or pre-processed 3D HRT geometry, or an existing CFPD simulation — and directly proceed to the Monte Carlo simulation. Based on experience within the research group, preparing a geometry and the input files for a CFD/CFPD simulation can require 1–2 working days if performed manually, with the outlet/inlet segmentation being the most time-consuming component. Using the presented framework, total preparation time was reduced to approximately 30 min, enabling the user to generate simulation-ready inputs directly from CT data. The CFD/CFPD simulation may require several hours or days, depending on model complexity, mesh resolution, and solver settings, all of which lie outside the current study scope. The authors did not find it informative to provide a specific processing time for each step of the pipeline since it heavily depends on computational resources, given the strong dependence on computational resources, CT dataset size, and mesh repair requirements.

While the workflow was tested in over 20 geometries, including geometries that encompassed the TB and ET regions individually and in combination, successfully converging a CFD/CFPD simulation, these cases may not capture the full variability encountered across diverse subject anatomies. Ongoing validation across broader HRT geometries is planned to refine alignment procedures and mesh robustness.

Several aspects of the pipeline remain areas for future refinement. A primary objective is to expand the framework's capacity to process complete HRT geometries in an automated manner, with particular focus on enhancing the integration of upper and lower airway segments from distinct CT datasets. Replacing rigid alignment with deformable image registration techniques will be investigated to improve the spatial mapping of CFPD-derived PDPs to mesh-based computational phantoms. Additional efforts will also aim to scale the pipeline for large-cohort studies (i.e., >50 subjects), incorporating reduced-order models to accelerate CFPD simulation while retaining anatomical fidelity. These extensions will support broader applications in respiratory health, including environmental exposure studies and occupational hazard assessments, with improved throughput and scalability.

## Figures and Tables

**Table 1:** Statistics for the voxel dimensions of the CT scans used in this work.

	x	y	z
Mean [mm]	0.67	0.67	0.95
Standard Dev. [mm]	0.07	0.07	0.17
Min [mm]	0.55	0.55	0.60
Max [mm]	0.78	0.78	1.25

**Table 2:** Key non-default parameters for SnappyHexMesh dict in OpenFOAM.

Parameter group	Parameter	Value
Main meshing steps	addLayers	True
Castellated mesh controls	maxLoadUnbalance	0.10
	nCellsBetweenLevels	3
Feature and surface refinement	feature refinement level	4
	min/max refinement level (airway outlets)	(2, 5)
	min/max refinement level (volume)	(4, 10)
Region-based refinement	refinement mode	Distance
	refinement levels	(0.0006, 4)
		(0.002, 3)
		(0.01, 2)
	resolveFeatureAngle	10
	allowFreeStandingZoneFaces	True
Snap controls	nSmoothPatch	3
	tolerance	4.0
	nSolveIter	400
	nRelaxIter	5
	nFeatureSnapIter	150
	explicitFeatureSnap	True
Layer addition controls	nSurfaceLayers (volume)	6
	expansionRatio	1.3
	finalLayerThickness	0.6
	minThickness	0.3
	featureAngle	80
	nRelaxIter	50
	nSmoothSurfaceNormals	5
	nSmoothNormals	6
	nLayerIter	200

**Table 3:** List of models, parameters, and boundary conditions for the airflow and particles in the simulations presented in this work. A more detailed explanation of the selection of these parameters can be found in the Supplemental Information, CFPD Modeling section. *Source*: Modified from Bartol, Graffigna Palomba et al. (2024).

Description	Parameter value/Condition (Flow solver)	Description	Parameter value/Condition (Flow solver)
Turbulence model	k-ω SST LM	Wall condition for $\vec{u}$	No slip boundary condition
Inlet boundary conditions for $\vec{u}$	Variable flow rate: $Q=Q_{\text{max}}\text{sin}\left(\frac{2\pi t}{T}\right)$	Airflow density and temperature	1.177 kg/m^3^; 300 K
Outlet boundary conditions $\vec{u}$	Zero gradient	Particle solver	MPPICFoam (OpenFOAM); Lagrangian multi phase (StarCCM+)
Inlet boundary conditions for p	Zero gradient	Particle coupling	MPPICFoam (OpenFOAM); Lagrangian
Outlet boundary conditions for p	Fixed pressure value (static pressure of the environment)	Particle distribution density function:	User-defined
Inlet boundary conditions for k	$\frac{3}{2}\left(I{\left(\left\|u_{\text{ref}}\right\|\right)}^{2}\right)$ I=0.04, 4% turbulence intensity	Particle density	User-defined
Inlet boundary conditions for ω	$\omega =\frac{k^{0.5}}{C_{\mu }^{0.25}L}$	Number of particles injected to the system	at least 100,000 particles (OpenFOAM)
Inlet boundary conditions for γ	γ=1	Injection rate	1/(time step) per injector point (StarCCM+)
Inlet boundary conditions for $Re_{\theta }$	See manuscript for full description, CFPD modeling	Particle-wall interaction mode	Stick upon contact with the wall
Outlet boundary conditions for $\gamma ,Re_{\theta },k,\omega$	Zero gradient	Time integration scheme	PISO algorithm (OpenFOAM); Implicit unsteady (StarCCM+)
Wall treatment for k	All $y^{+}$ treatment (StarCCM+); kLowReWallFunction (OpenFOAM)	Time step used	0.015 s–0.025 s
Wall treatment for ω	All $y^{+}$ treatment (StarCCM+); omegaWallFunction (OpenFOAM)	Total time simulated	4 s. (2 s. flow only and 2 s. with particles)

**Table 4:** Organ dose comparison between uniform particle distribution assumption and CFPD-informed particle distribution. The PHITS simulation parameters were modeled for I^131^ with a total activity of 17.48 Bq, partitioned as 8.742 Bq in the BB region and 8.735 Bq in the trachea.

Organ	Volume (cm^3^)	CFPD dose (Gy/Source)	Relative error (%)	Uniform dose (Gy/Source)	Uniform relative error (%)	Relative dose difference (%)
Lung right	1342.1	3.82 × 10^−13^	0.01782	4.78 × 10^−14^	0.090	−77.74
Lung left	1123.8	2.04 × 10^−13^	0.02836	3.38 × 10^−14^	0.110	−71.57
Trachea	10.038	5.58 × 10^−13^	0.12951	2.73 × 10^−11^	0.035	95.99
BB	2.643	6.56 × 10^−12^	0.18992	6.12 × 10^−10^	0.018	97.88
Liver	1800.9	5.05 × 10^−15^	0.08027	6.93 × 10^−15^	0.130	15.63
Stomach	151.538	1.63 × 10^−14^	0.43730	2.28 × 10^−14^	0.708	16.81
Bladder	39.743	1.31 × 10^−16^	6.02034	1.69 × 10^−16^	5.938	12.71
Small intestine	672.853	2.72 × 10^−15^	0.66355	3.69 × 10^−15^	1.027	15.01
Ascending colon	93.548	1.64 × 10^−15^	2.62257	2.06 × 10^−15^	3.432	11.46
Descending colon	93.548	4.20 × 10^−15^	2.21698	5.59 × 10^−15^	4.050	14.22
Sigmoid colon	41.576	3.47 × 10^−16^	6.38531	4.52 × 10^−16^	7.766	13.15
Transverse colon	124.731	6.42 × 10^−15^	0.98471	9.27 × 10^−15^	1.720	18.21

**Fig. A.17.**
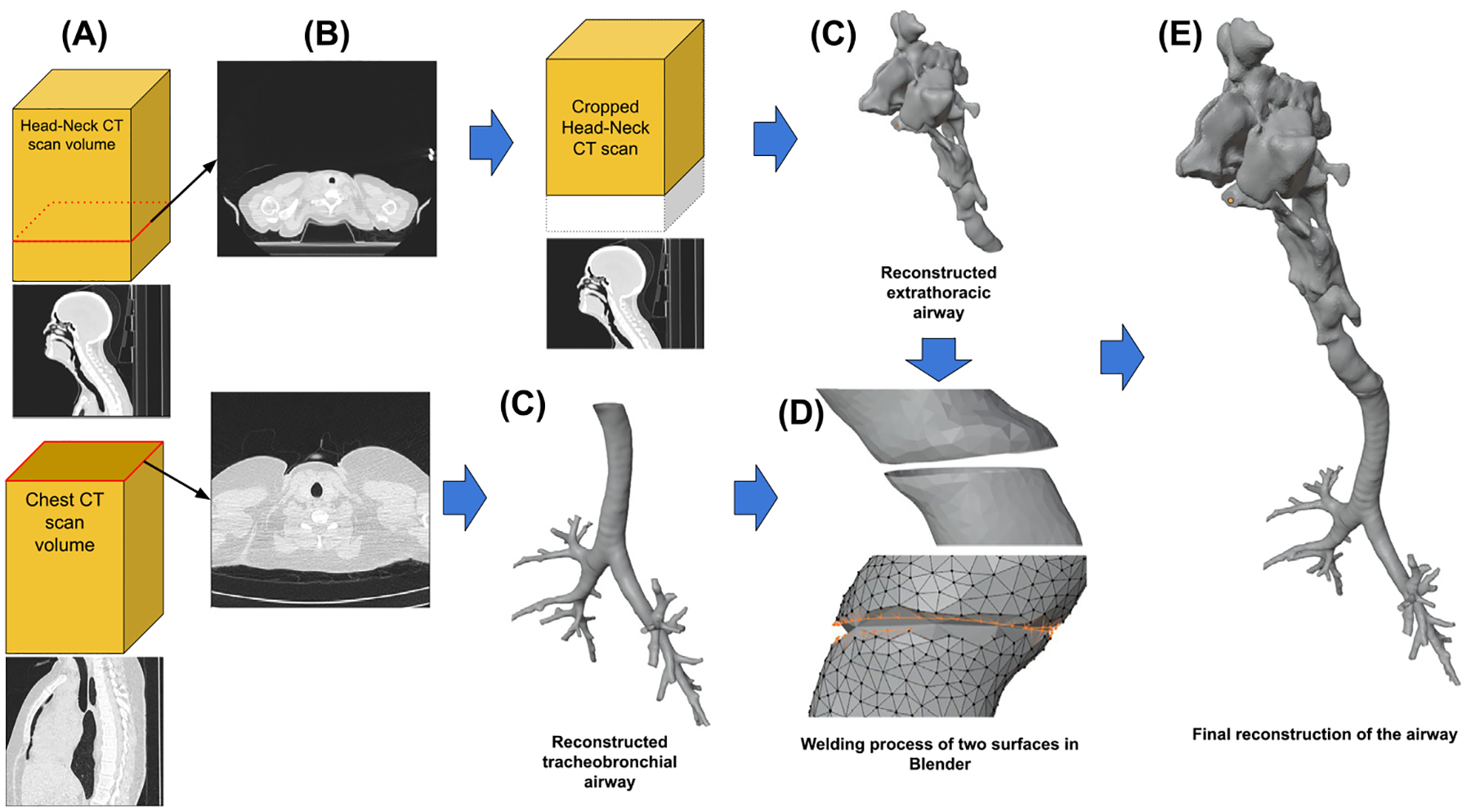
Visual steps of the manual blending procedure: (A) Head-neck and chest CT scans initially acquired; (B) Identification of anatomically matched slices and cropping excess slices; (C) Independent segmentation of extrathoracic and tracheobronchial regions; (D) Manual welding of the two segments using Blender; (E) Final integrated 3D airway geometry.

## Data Availability

A permanent repository for CFPD ready geometries is available at URL: https://dx.doi.org/10.6084/m9.figshare.24787773 (Bartol, Graffigna et al., 2024). All the other relevant data are available from the corresponding author upon reasonable request.The CT scan databases used were from publicly available databases. A link to the data can be found in the manuscript.

## Code availability

Although the core source code is not intended for open-source release, a GUI software enabling users to upload either CT scans or raw 3D geometries into the framework will be made available in the future at: https://github.com/ignaciobartol/CT-to-CFD-airways.

## Appendix. Manual blending procedure for combining tracheobronchial and extrathoracic HRT segments

The process of blending the extrathoracic and tracheobronchial regions into a single 3D geometry it must be manually done. Although automation could be feasible with the appropriate datasets, the current public available full-body CT scan datasets feature a very thick slice thickness (2.34 mm in the dataset Selfridge et al., 2023 from TCIA, in the work by Sundar et al., 2022), making not feasible to obtain a segmentation of either the TB or ET regions with the current algorithms.

To address this limitation, matching subjects were manually identified across the separate head-neck and chest CT scan datasets based solely on age and sex, as this was the only available demographic information. Following the identification of appropriate subject pairs, the images were visually analyzed to identify anatomical landmarks, specifically around the lower larynx or upper trachea, to align the transition between the ET and TB regions. Any excess slices in the head-neck scans were removed once alignment was visually confirmed, and the modified image was then segmented using the upper respiratory tract algorithm.

After obtaining separate segmentations for the ET and TB airway regions, Blender (Community, 2018) software's welding tool was utilized to blend the two segmented surfaces into a cohesive 3D airway model. This manual welding involved aligning vertices and edges to ensure continuity and smooth transitions between the two airway segments. Remeshing could be done or not depending on the final quality of the blending. Fig. A.17 visually summarizes the key steps involved in this manual blending process.
